# Deciphering gene–smoking interactions in age-related macular degeneration through cross-biobank genomic integration

**DOI:** 10.18332/tid/205419

**Published:** 2025-07-29

**Authors:** Ju Guo, Yuhan Jiang, Xinran Xu, Jianhua Wang, Xueming Yao, Xiaohong Wang, Hongxi Yang, Mulin J. Li, Hua Yan

**Affiliations:** 1 Department of Ophthalmology, Ministry of Education International Joint Laboratory of Ocular Diseases, Tianjin Key Laboratory of Ocular Trauma, Tianjin Institute of Eye Health and Eye Diseases, China-UK ‘Belt and Road’ Ophthalmology Joint Laboratory, Tianjin Medical University General Hospital, Tianjin, China; 2 Department of Pulmonology, Tianjin Children’s Hospital (Children’s Hospital of Tianjin University), Tianjin, China; 3 Department of Bioinformatics, The Province and Ministry Co-sponsored Collaborative Innovation Center for Medical Epigenetics, School of Basic Medical Sciences, Tianjin Medical University, Tianjin, China; 4 Department of Pharmacology, Tianjin Key Laboratory of Inflammation Biology, School of Basic Medical Sciences, Tianjin Medical University, Tianjin, China

**Keywords:** polygenic risk score, ever smoked, prs–smoking interactions, variant–smoking interactions, complement pathway

## Abstract

**INTRODUCTION:**

This study aims to identify genetic loci associated with age-related macular degeneration (AMD) and assess the interaction between genetic susceptibility and smoking history.

**METHODS:**

A meta-analysis of discovery genome-wide association studies (GWASs), involving a total of 42542 AMD patients and 920322 controls from four large-scale European cohorts, was conducted using METAL, a software tool commonly used for meta-analysis of GWAS. A polygenic risk score (PRS) was derived from the meta-analysis results for 331281 UK Biobank participants. Cox proportional hazards models evaluated interactions between genetic predisposition and smoking history at both PRS and variant levels. Logistic regression models examined plasma complement protein profiles across AMD PRS and smoking status groups.

**RESULTS:**

We identified two novel risk loci, OCA2 melanosomal transmembrane protein (OCA2) and nitric oxide associated 1 (NOA1). Incorporating the PRS significantly enhanced AMD risk prediction in 331281 UK Biobank participants, with the area under the curve (AUC) increasing from 0.74 to 0.76 (p=2×10^-16^). During a mean follow-up of 13.6 years, Cox models revealed significant additive (relative excess risk due to interaction, RERI=0.13; 95% CI: 0.06–0.19; attributable proportion, AP=0.08; 95% CI: 0.04–0.13; synergy index, SI=1.33; 95% CI: 1.13–1.56) and multiplicative interactions (hazard ratio, HR=1.08; 95% CI: 1.03–1.14, p=2.65×10^-3^) between PRS and smoking history. Variant-level interactions were prominent at complement factor H (CFH) and complement factor I (CFI) loci. Individuals who have ever smoked and high PRS exhibited dysregulated plasma proteins in the alternative, classical and lectin complement pathways.

**CONCLUSIONS:**

This study revealed the genetic architecture of AMD and highlighted the synergistic effects of smoking and genetic risk, emphasizing the potential need to integrate genetic assessments into prevention strategies.

## INTRODUCTION

Age-related macular degeneration (AMD) is the leading cause of significant vision impairment among individuals aged >55 years in developed countries, accounting for 6–9% of global cases of legal blindness. By 2040, the number of people affected by AMD worldwide is projected to reach approximately 288 million^1^. The onset of AMD is influenced by a complex interplay of aging, environmental risk factors, and genetic predisposition, with dozens of genetic risk factors having been well established^2^. Among non-genetic contributors, both aging and smoking are consistently recognized as significant risk factors^3^. Notably, smoking has long been identified as one of the most modifiable risk factors for AMD, with studies showing a clear association between tobacco use and an increased risk of disease progression. Genetic predisposition also plays a crucial role in AMD risk, particularly genetic variants in the complement pathway related locus, such as CFH, CFI and C3 gene^4-6^, being strongly associated with susceptibility to the disease. Gene-environment interactions involving variations in the CFH gene and smoking have been linked to an even greater risk of AMD, highlighting the combined influence of genetic factors and environmental exposures^7^.

However, the generalizability of these findings remains limited, underscoring the necessity for larger prospective studies to elucidate these associations. The UK Biobank (UKBB) is a unique resource that provides high-quality, large-scale genotype and phenotype data, facilitating in-depth analyses in epidemiological research^8^. It offers a robust platform that enhances the understanding of complex disease mechanisms and advances the field of precision medicine. This resource not only improves the accuracy and reliability of epidemiological studies but also provides significant opportunities to uncover the interactions between genetic predisposition and environmental influences. Although studies on polygenic risk score (PRS)–smoking interactions have been widely conducted for diseases such as lung cancer^9,10^, COPD^11^, and cardiovascular disease^12^, they remain relatively rare in the context of AMD.

In this study, we leveraged large-scale genomic data from multiple biobanks to conduct a genome-wide association study (GWAS) meta-analysis and identify genetic risk variants associated with AMD. Based on these variants, we constructed PRS and subsequently assessed the interaction between genetic susceptibility and smoking history using both the PRS and individual genetic variants.

## METHODS

### Study design and population

This study has been reported in accordance with the STROCSS 2024 guidelines^13^. The study adhered to the principles outlined in the Declaration of Helsinki. Ethics approval was secured from the pertinent authorities, including the North West Multi-Centre Research Ethics Committee for UK Biobank (approval number 11/NW/0382).

**Figure 1:**
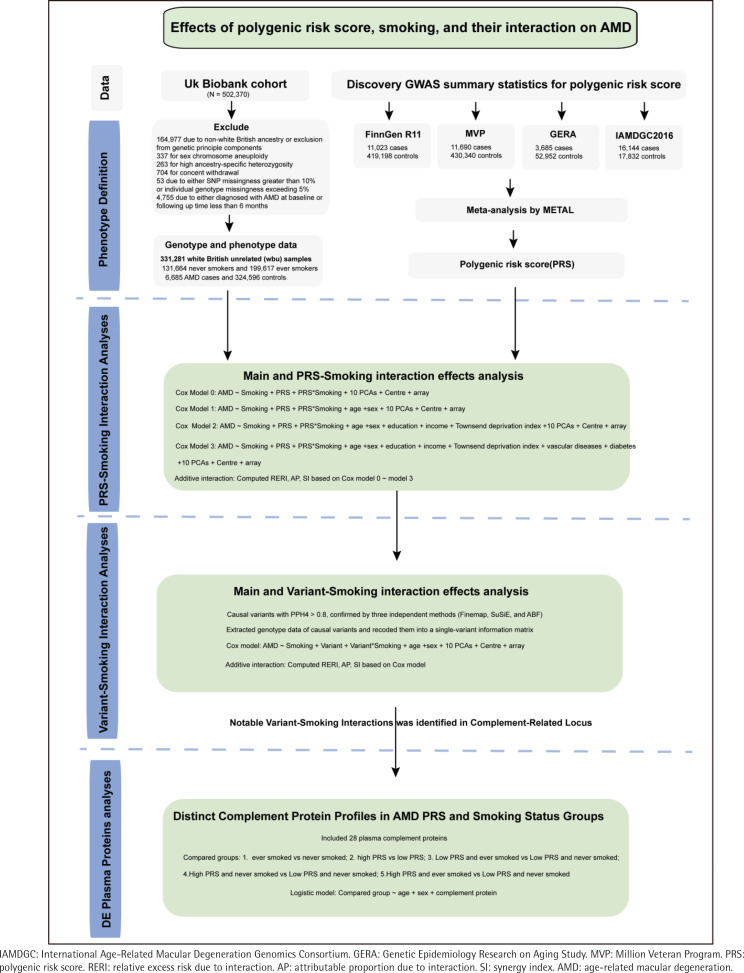
Overview of study design

The UKBB constitutes a large-scale prospective cohort study involving >500000 participants aged 38–73 years, recruited between 2006 and 2010. Participant data encompass genome-wide genotyping, medical history, lifestyle factors, plasma protein levels, blood and urine biomarkers, as well as physical and anthropometric measurements^8^. As illustrated in Figure 1, we performed a meta-analysis of GWAS using four recent, large-scale AMD datasets from independent European cohorts external to the UKBB. Leveraging phenotype and genotype data from 331281 White European participants in the UKBB, we constructed PRS and assessed the effects of PRS, smoking history (ever smoked), their interaction, and variant–smoking interactions on the AMD incidence. Furthermore, we examined dysregulated plasma proteins across AMD PRS and smoking status groups to pinpoint specific pathways or proteins that are affected by both genetic and environmental factors, shedding light on the how these factors jointly influence AMD risk.

After excluding 164977 participants for non-White British ancestry or genetic principal component considerations to ensure ancestral consistency, 337 for sex chromosome aneuploidy, 263 for elevated heterozygosity, 704 for withdrawal of consent, 53 for SNP missingness greater than 1% or individual genotype missingness exceeding 5%, and 4755 due to a diagnosis of AMD with a follow-up period of less than six months. Consequently, 331281 unrelated White British individuals were included in the final analysis. Detailed information on the inclusion and exclusion criteria is shown in the Supplementary file Methods and Supplementary file Figure 1.

### Discovery GWAS summary statistics and meta-analysis

We conducted a meta-analysis of genome-wide association studies (GWASs) from 4 independent European cohorts, ensuring no sample overlap: the International Age-Related Macular Degeneration Genomics Consortium (IAMDGC) AMD-2016 GWAS (16144 patients with advanced AMD and 17832 control participants)^4^; the FinnGen R11 AMD GWAS (16144 patients with advanced AMD and 17832 control participants)^14^; the Genetic Epidemiology Research on Aging (GERA) study (3685 AMD cases and 52952 controls)^15^; and the Million Veteran Program (MVP) study (11690 AMD cases and 430340 controls)^16^. We performed a sample size-weighted fixed-effect meta-analysis of the discovery GWASs utilizing METAL^17^.

### Fine-mapping of meta-AMD GWAS

To identify the likely causal genetic variants within the regions associated with meta-AMD GWAS signals, we employed three Bayesian fine-mapping algorithms: Approximate Bayes Factor (ABF)^18^, FINEMAP^19^, and Sum of Single Effects (SuSiE)^20^. Posterior probabilities (PP) were computed for each variant, and those with a PP>0.8, consistently identified by all three methods, were designated as causal variants for each locus (further details are provided in the Supplementary file Methods).

### Replication in the UK Biobank datasets

To replicate the risk loci identified in our meta-analysis of AMD GWAS, we conducted a GWAS using high-quality genotype data from 331281 individuals in the UKBB. The analysis was performed utilizing the REGENIE framework^21^, with covariates including age, sex, age-squared, the interaction between age and sex (age × sex), UKBB assessment center (22 categories), genotyping batch, and the first 20 principal components. Subsequently, we extracted the causal variants identified through fine-mapping and assessed the effect sizes in both the meta-AMD GWAS and UKBB AMD GWAS. A Spearman correlation analysis was conducted using the base functions in R (version 4.3.3) to evaluate the consistency of effect sizes across the two datasets.

### Polygenic risk score for AMD

To construct polygenic risk scores (PRSs), we filtered variants to retain only HapMap3 SNPs^22^ with an INFO score >0.9, meta-AMD GWAS MAF <0.01, and MAF discrepancies between the meta-AMD GWAS and LD reference panels <0.2. PRS was generated using PRS-Continuous Shrinkage (PRS-CS, version 3 Nov 2022)^23^ with a default global shrinkage prior of 1×10^-4^, 1000 Markov Chain Monte Carlo (MCMC) iterations, and 500 burn-in iterations, leveraging an LD reference panel from the 1000 Genomes Project. The default shrinkage parameter has been extensively validated and is recommended for large-scale datasets with European ancestry, due to its robust performance in minimizing overfitting while maintaining predictive accuracy. Posterior SNP effect sizes were estimated, and per-individual PRS was calculated as the genome-wide sum of posterior SNP effect sizes weighted by allele dosage, using PLINK version 1.90p (64-bit, 11 Dec 2023)^24^. To evaluate the incremental predictive utility of the AMD PRS, we employed complementary statistical approaches: 1) the DeLong test to assess the statistical significance of AUC differences between baseline and PRS-enhanced models; and 2) 1000 bootstrap iterations to quantify the stability of observed AUC improvements, with full methodological details provided in the Supplementary file Methods.

### PRS–smoking interaction models

To evaluate the association between smoking, PRS, their interaction, and AMD events, we employed multivariable Cox proportional hazards regression models, implemented through the R package *survival* (version: 3.7.0). Multiplicative interaction was assessed based on the hazard ratios (HRs) and p-values of the PRS × smoking interaction term in the multivariable Cox models. Additive interaction was evaluated using three key parameters: the relative excess risk due to interaction (RERI), attributable proportion due to interaction (AP), and the synergy index (SI), all derived from the multivariable Cox models. Definitions of multiplicative interaction, additive interaction, AMD and smoking history, as well as the detailed information about PRS–smoking interaction models are provided in the Supplementary file Methods.

### Variant–smoking interaction models

To elucidate which AMD risk loci are predominantly involved in the PRS–smoking interaction, we conducted variant–smoking interaction analyses, focusing on causal variants identified in the meta-AMD GWAS. Similarly, multivariable Cox proportional hazards models were applied to assess the association between smoking, individual variants, their interaction, and AMD incidence. Additive interaction metrics (RERI, AP, and SI) were computed based on the variant–smoking interaction Cox models. For significant interactions, chi-squared tests (using base R functions) were performed to compare the relative risk between individuals who have smoked and are homozygous for risk variants, compared with those who have never smoked and do not carry these variants. Detailed information about variant–smoking interaction models are provided in the Supplementary file Methods.

### Distinct plasma complement proteins in AMD PRS and smoking status groups

Motivated by significant variant–smoking interactions observed at complement-related loci, we examined the expression profiles of 28 complement pathway proteins in relation to disease status, PRS, and smoking status. Protein names (Supplementary file Table S1) were sourced from the Kyoto Encyclopedia of Genes and Genomes (KEGG) database^25^, with expression data from the UKBB dataset.

Participants were categorized into groups based on their PRS and smoking status. Smoking status was classified into two categories: never smoked and ever smoked. PRS was stratified into ‘low’ and ‘high’ groups using the median of the PRS distribution as the cut-off point. Additionally, combinations of PRS and smoking status were used to define distinct subgroups, enabling a comprehensive evaluation of their joint effects on AMD risk. Due to the large sample sizes, traditional differential expression methods were insufficient, therefore logistic regression, adjusted for age, sex, body mass index (BMI, Data-Field 21001), alcohol drinker status (Data-Field 20117), vascular/heart problems diagnosed by doctor (Data-Field 6150), diabetes diagnosed by doctor (Data-Field 2443), medication for cholesterol, blood pressure or diabetes (Data-Field 6177, male), medication for cholesterol, blood pressure, diabetes, or take exogenous hormones (Data-Field 6153, female), was performed using the R package caret (version 6.0.94). Five key comparisons were made: ever smoked versus never smoked, high PRS versus low PRS, low PRS with ever smoked versus low PRS with never smoked, high PRS with never smoked versus low PRS with never smoked, and high PRS with ever smoked versus low PRS with never smoked. Results are presented as odds ratios (ORs) with 95% confidence intervals (CIs) and p-values.

### Sensitive analyses

To ensure the robustness and generalizability of our models, we conducted a series of sensitivity analyses as provided in the Supplementary file Methods. We applied standard genome-wide significance thresholds (p<5×10^-8^) for the GWAS meta-analysis. For the PRS–smoking interaction, as well as variant–smoking interactions, the focus was specifically on a single environmental exposure-smoking status. Therefore, statistical significance in interaction models was defined as p<0.05, with highly significant results at p<0.01. For the complement protein analysis, where multiple proteins were tested, we applied Bonferroni correction to control for type I error. Specifically, statistical significance for the protein expression analysis was defined as p<0.0018 (0.05/28). To account for multiple testing in the Mendelian randomization (MR) analyses conducted as sensitivity analyses (described in the Supplementary file Methods), we applied the False Discovery Rate (FDR) correction using the Benjamini–Hochberg (BH) procedure.

## RESULTS

### Meta-analysis of GWAS for AMD and replication in the UK Biobank

In total, 79 independent genome-wide significant SNPs were identified across 41 well-established AMD loci (such as CFH, ARMS2, C3, and VEGFA), along with two novel index SNPs (rs1713998 and rs12913832) near or within NOA1 and OCA2, respectively, reaching genome-wide significance for the first time (p<5×10^-8^) (Supplementary file: Figure 1 and Table S2).

Fine-mapping analyses identified 21 potential causal variants with PP>0.8, confirmed by three independent methods (FINEMAP, SuSiE, and ABF) (Supplementary file Table S3). We subsequently replicated these 21 variants in the UKBB dataset (6685 cases and 324596 controls), demonstrating high concordance of SNP effect sizes between the meta-analysis and UKBB replication (Spearman’s correl a tion coeff i cient 0.88, p=8.3×10^-7^, Supplementary file Figure 2). Of the 21 loci, 13 were nominally significant (p<0.05) in UKBB, including one variant (rs4388642 in CFH, p=1.82×10^-17^) reaching Bonferroni-corrected significance (Supplementary file Table S3).

### Polygenic risk score and AMD risk prediction

The characteristics of the 331281 UKBB participants are summarized in Table 1. The AMD PRS was strongly associated with AMD status in the UKBB validation cohort (OR=1.41; 95% CI: 1.38–1.43, p<2×10^-16^, logistic regression), with an AUC of 0.59 (95% CI: 0.59–0.60). To benchmark the PRS against traditional AMD risk factors, we established a null model using age, sex, smoking status, and the top 10 PCs (AUC=0.74; 95% CI: 0.74–0.75). Adding the AMD PRS to this model significantly improved the AUC to 0.76 (95% CI: 0.75–0.76; Z=11.90, p=2×10^-16^, Supplementary file Figure 3). Bootstrap resampling further validated the stability of this improvement, showing a mean ΔAUC of 0.01 (95% CI: 0.01–0.02; Supplementary file Figure 4).

**Table 1:** Baseline characteristics of participants

*Characteristics*	*Patients (N=6685)* *n (%)*	*Control participants (N=324596)* *n (%)*	*Total* *n*
**Sex**			
Male	2623 (39.2)	150686 (46.4)	153309
Female	4062 (60.8)	173910 (53.6)	177972
**Age** (years), mean ± SD	63 ± 5	57 ± 8	57 ± 8
**Education level**			
College or university degree	1645 (24.6)	102887 (31.7)	104532
A levels/AS levels or equivalent	1352 (20.2)	88889 (27.4)	90241
O levels/GCSEs or equivalent	2787 (41.7)	154401 (47.6)	157188
CSEs or equivalent	381 (5.7)	43429 (13.4)	43810
NVQ or HND or HNC or equivalent	975 (14.6)	60898 (18.8)	61873
Other professional qualifications (e.g. nursing, teaching)	1766 (26.4)	94173 (29.0)	95939
**Income** (£)			
<18000	1916 (28.7)	60112 (18.5)	62028
18000–30999	1672 (25.0)	71655 (22.1)	73327
31000–51999	1164 (17.4)	74550 (23.0)	75714
52000–100000	623 (9.3)	58663 (18.1)	59286
>100000	120 (1.8)	15236 (4.7)	15356
**Ever smoked**			
Yes	4224 (63.2)	195393 (60.2)	199617
No	2461 (36.8)	129203 (39.8)	131664
**Alcohol drinker status**			
Never	285 (4.3)	9878 (3.0)	10163
Previous	268 (4.0)	10983 (3.4)	11251
Current	6127 (91.7)	303486 (93.5)	309613
**Socio-economic status quintile**			
1 (least deprived)	1341 (20.0)	65135 (20.1)	66476
2–4	4008 (60.0)	194579 (59.9)	198587
5 (most deprived)	1336 (20.0)	64882 (20.0)	66218
**BMI** (kg/m^2^)	27.83 ± 4.85	27.38 ± 4.74	27.39 ± 4.74
**Vascular/heart problems diagnosed by doctor**			
Yes	2045 (30.6)	100174 (30.9)	102219
No	4634 (69.3)	224036 (69.0)	228670
**Diabetes diagnosed by doctor**			
Yes	577 (8.6)	14829 (4.6)	15406
No	6089 (91.0)	309081 (95.2)	315170

To assess the capacity of model for reclassifying individuals based on predicted risk, we computed the NRI and IDI metrics. To fortify the reliability and robustness of the findings, we applied bootstrap resampling to evaluate the stability of the results across datasets. The categorical NRI was 0.085 (95% CI: 0.075–0.096, p<2×10^-16^) and the continuous NRI was 0.267 (95% CI: 0.243–0.291, p<2×10^-16^), both indicating substantial improvements in risk reclassification. Furthermore, the IDI was 0.48% (95% CI: 0.45–0.52, p<2×10^-16^), further reinforcing the value of adding PRS to traditional risk factors in AMD prediction.

To evaluate the stratification performance of PRS, we selected 20% of UKBB individuals with high PRS (n=66259) and 20% with low PRS (n=66257). The overall AMD prevalence in the UKBB cohort was approximately 2.0%, compared to 3.3% in the high-PRS group and 1.4% in the low-PRS group. Cumulative incidence analysis revealed that for individuals aged >70 years, the AMD incidence was 19.5 ± 0.6% in the entire cohort, rising to 31.7 ± 1.6% in the high-PRS group, compared to 12.5 ± 1.1% in the low-PRS group, and 17.4 ± 0.7% in the mid-PRS group (Supplementary file Figure 5).

### Effects of polygenic risk score, ever smoked, and their interaction on AMD

During a mean follow-up period of 13.6 years (range: 0.5–16.7 years), 6685 participants developed AMD. In this prospective cohort study, ever smoked was significantly associated with a higher risk of AMD in both Model 0 (HR=1.12; 95% CI: 1.06–1.17, p=4.73×10^-5^) and Model 1 (HR=1.06; 95% CI: 1.01–1.12, p=2.02×10^-2^). Although the association was borderline significant in Model 2 (HR=1.05; 95% CI: 1.00–1.11, p=0.07), it was not significant in Model 3 (HR=1.04; 95% CI: 0.98–1.10, p=0.17). PRS consistently showed a strong positive association with AMD risk across all models. The smallest effect size was seen in Model 0 (HR=1.34; 95% CI: 1.29–1.39, p<2×10^-16^), while the largest occurred in Model 2 (HR=1.35; 95% CI: 1.29–1.40, p<2×10^-16^).

**Figure 2:**
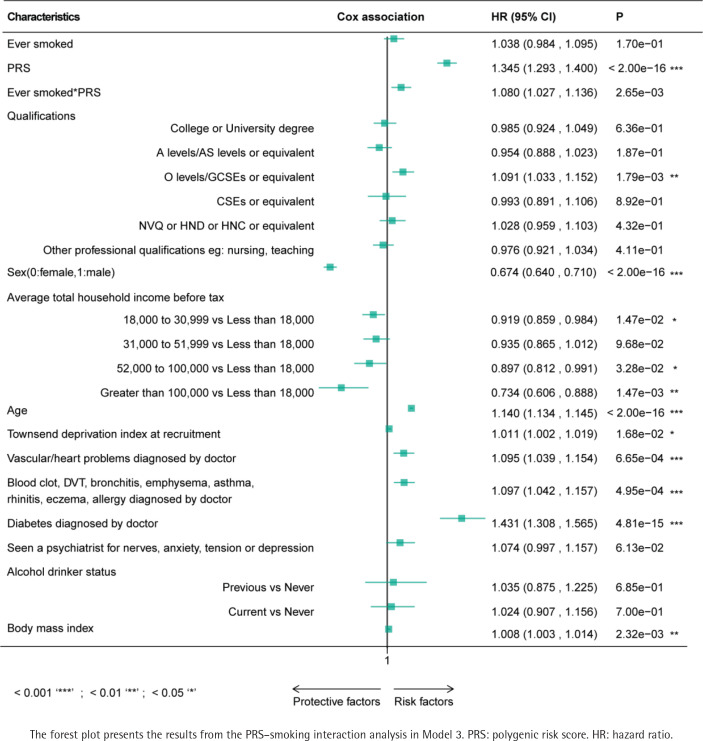
Interaction between PRS and smoking in Model 3

Significant positive additive and multiplicative interactions between smoking status (ever smoked) and PRS were observed across all models (Table 2). The strongest interaction effects were found in Model 0 (RERI=0.15; 95% CI: 0.08–0.22, AP=0.10; 95% CI: 0.05–0.14, SI=1.34; 95% CI: 1.16–1.54, and a multiplicative HR=1.08; 95% CI: 1.02–1.13; p=3.95×10^-3^). In the most complex model (Model 3), the covariates including female sex, age, vascular or heart diseases, allergic diseases, diabetes, body mass index, and education level (O levels/GCSEs or equivalent qualifications) were identified as potential positive risk factors for AMD (p<0.05, Figure 2).

**Table 2:** Effects of polygenic risk score, ever smoked, and their interaction on AMD

*Models*	*Ever smoked*	*PRS*	*RERI*	*AP*	*SI*	*Multiplicative interaction*
**Full**						
**Model 0**						
HR (95% CI)	1.12 (1.06–1.17)	1.34	0.15 (0.08–0.22)	0.10 (0.05–0.14)	1.34 (1.16–1.54)	1.08 (1.02–1.13)
p	4.73×10^-5^	<2×10^-16^				3.95×10^-3^
PH_p	0.28					
C-index (SE)	0.61 (0.004)					
**Model 1**						
HR (95% CI)	1.06 (1.01–1.12)	1.35	0.14 (0.07–0.21)	0.09 (0.05–0.13)	1.33 (1.14–1.55)	1.08 (1.03–1.14)
p	2.02×10^-2^	<2×10^-16^				2.73×10^-3^
PH_p	0.30					
C-index (SE)	0.76 (0.003)					
**Model 2**						
HR (95% CI)	1.05 (1.00–1.11)	1.35	0.13 (0.06–0.20)	0.09 (0.04–0.13)	1.33 (1.14–1.56)	1.08 (1.03–1.14)
p	6.59×10^-2^	<2×10^-16^				2.70×10^-3^
PH_p	0.30					
C-index (SE)	0.76 (0.003)					
**Model 3**						
HR (95% CI)	1.04 (0.98–1.10)	1.35	0.13 (0.06–0.19)	0.08 (0.04–0.13)	1.33 (1.13–1.56)	1.08 (1.03–1.14)
p	1.70×10^-1^	<2×10^-16^				2.65×10^-3^
PH_p	0.30					
C-index (SE)	0.76 (0.003)					
**Test**						
**Model 0**						
HR (95% CI)	1.10 (1.02–1.18)	1.35	0.15 (0.05–0.24)	0.09 (0.03–0.15)	1.33 (1.09–1.62)	1.08 (1.00–1.15)
p	1.25×10^-2^	<2×10^-16^				4.34×10^-2^
PH_p	0.15					
C-index (SE)	0.61 (0.01)					
**Model 1**						
HR (95% CI)	1.10 (1.02–1.19)	1.34	0.12 (0.03–0.22)	0.08 (0.02–0.14)	1.28 (1.04–1.57)	1.06 (0.99–1.14)
p	9.55×10^-3^	<2×10^-16^				0.11
PH_p	0.99					
C-index (SE)	0.75 (0.004)					
**Model 2**						
HR (95% CI)	1.03 (0.95–1.11)	1.37	0.10 (-0.001–0.19)	0.06 (0.001–0.13)	1.24 (0.99–1.55)	1.06 (0.99–1.14)
p	0.47	<2×10^-16^				0.11
PH_p	0.07					
C-index (SE)	0.75 (0.004)					
**Model 3**						
HR (95% CI)	1.04 (0.96–1.12)	1.32	0.13 (0.03–0.22)	0.09 (0.02–0.15)	1.36 (1.06–1.73)	1.08 (1.01–1.16)
p	0.36	<2×10^-16^				0.03
PH_p	0.06					
C-index (SE)	0.76 (0.004)					
**Train**						
**Model 0**						
HR (95% CI)	1.13 (1.05–1.22)	1.33	0.16 (0.06–0.26)	0.10 (0.04–0.16)	1.35 (1.11–1.64)	1.08 (1.00–1.16)
p	0.001	<2×10^-16^				0.04
PH_p	0.95					
C-index (SE)	0.60 (0.01)					
**Model 1**						
HR (95% CI)	1.03 (0.95–1.11)	1.36	0.15 (0.05–0.25)	0.10 (0.04–0.16)	1.39 (1.11–1.75)	1.10 (1.03–1.18)
p	0.50	<2×10^-16^				7.77×10^-3^
PH_p	0.150					
C-index (SE)	0.76 (0.004)					
**Model 2**						
HR (95% CI)	1.08 (1.0–1.16)	1.32	0.17 (0.07–0.27)	0.11 (0.05–0.17)	1.42 (1.14–1.77)	1.10 (1.03–1.18)
p	5.53×10^-2^	<2×10^-16^				8.11×10^-3^
PH_p	0.73					
C-index (SE)	0.76 (0.004)					
**Model 3**						
HR (95% CI)	1.04 (0.96–1.12)	1.37	0.12 (0.03–0.22)	0.08 (0.02–0.14)	1.3 (1.05–1.62)	1.08 (1.003–1.16)
p	0.3	<2×10^-16^				0.04
PH_p	0.64					
C-index (SE)	0.76 (0.004)					
**Sensitivity**						
**analysis**						
**Model 3 in full**						
**cohort**						
HR (95% CI)	0.94 (0.87–1.01)	1.31	0.09 (0.03–0.16)	0.07 (0.03–0.12)	1.38 (1.10–1.73)	1.09 (1.04–1.15)
p	9.83×10^-2^	<2×10^-16^				2.19×10^-4^
PH_p	0.84					
C-index (SE)	0.76 (0.003)					

The PRS–smoking interaction models were conducted using Cox proportional hazards regression. Model 0: adjusts for the top 10 genetic principal components, UK Biobank assessment center and genotype measurement batch. Model 1: adjusts as in Model 0 plus age at exposure measurement and sex. Model 2: adjusts as in Model 1 plus socio-economic factors, including educational qualifications, average total household income before tax, and the Townsend deprivation index at recruitment. Model 3: adjusts as in Model 2 plus alcohol drinking status, body mass index (BMI), history of vascular or heart problems; diabetes, respiratory and allergic conditions such as blood clot, deep vein thrombosis (DVT), bronchitis, emphysema, asthma, rhinitis, eczema, or other allergies diagnosed by a doctor; mental health factors, such as having seen a psychiatrist for nerves, anxiety, tension, or depression. AMD: Age-Related Macular Degeneration. PRS: polygenic risk score. RERI: relative excess risk due to interaction. AP: attributable proportion due to interaction. SI: synergy index. HR: hazard ratio. PH_p: The p-value of proportional hazard assumption. SE: standard error.

Additionally, all models passed the proportional hazards (PH) assumption test (p>0.05). The C-index increased significantly with model complexity, from 0.60 in Model 0 to 0.76 in Model 3. Consistent results were observed in cross-validation and sensitivity analyses, which included individuals with AMD onset within six months of baseline. These findings further confirm the stability and robustness of our models, with consistently significant positive additive and multiplicative interactions.

**Figure 3:**
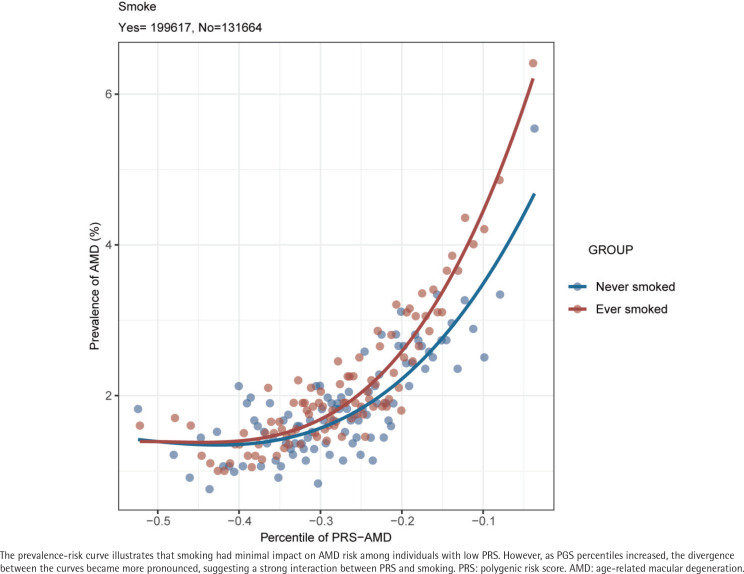
Smoking modifies the PRS-AMD prevalence-risk curve

The prevalence-risk curve in Figure 3 shows that ever smoking had little effect on AMD risk in individuals with low PRS, but as PRS percentiles increased, the divergence between curves became more pronounced, highlighting a strong PRS–smoking interaction.

### Notable variant–smoking interactions identified in complement-related locus

To identify which AMD risk loci are primarily involved in the PRS–smoking interaction, we performed variant–smoking interaction analyses focusing on 21 causal variants from the meta-AMD GWAS (Supplementary file Table S4).

As shown in Supplementary file Figure 6 (marked by the red lines in the forest plot), individuals carrying the rs4388642-C variant who had ever smoked exhibited both additive (RERI=0.1; 95% CI: 0.02–0.18, AP=0.08; 95% CI: 0.03–0.14) and multiplicative interactions (HR_mi_=1.08; 95% CI: 1.01–1.17; p=0.04). As shown in Table 3, homozygous carriers of rs4388642-CC who had ever smoked exhibited a significantly higher risk of AMD compared to rs4388642-TT carriers who had never smoked (OR=1.61; p=2×10^-16^). Additionally, heterozygous rs4388642-CT smokers had a relative risk of 1.26 (p=1.46×10^-9^). Conversely, the protective variant rs10922273-T in the CFH locus demonstrated negative interactions with smoking (RERI= -0.23; 95% CI: -0.36 – -0.09; HR_mi_=0.81; 95% CI: 0.71– 0.92; p=9.12×10^-4^). Neither homozygous nor heterozygous rs10922273-T carriers who had ever smoked exhibited a significant increase in AMD risk (p>0.05). Additionally, positive additive interactions were observed for the risk variant rs17562659-C (RERI=0.13; 95% CI: 0.0008–0.25, AP=0.10; 95% CI: 0.003–0.2). Both homozygous (OR=1.45, p=0.01) and heterozygous (OR=1.29; p=1.37×10^-9^) rs17562659-C carriers who had ever smoked exhibited a significantly increased risk of AMD compared to rs17562659-TT carriers who had never smoked.

**Table 3:** Effects of ever smoked and risk variant carriers on AMD

*Variant & smoking status*	*AMD*	*Controls*	*OR (95% CI)*	*χ^2^*	*p*
rs4388642-CC & ever smoked	531	18314			
rs4388642-TT & never smoked	1122	62464			
			1.61 (1.45–1.79)	81.52	2×10^-16^
rs4388642-CT & ever smoked	1877	82965			
rs4388642-TT & never smoked	1122	62464			
			1.26 (1.17–1.36)	36.59	1.46×10^-9^
rs17562659-CC & ever smoked	50	1825			
rs17562659-TT & never smoked	1973	104396			
			1.45 (1.09–1.93)	6.19	0.01
rs17562659-CT & ever smoked	836	34342			
rs17562659-TT & never smoked	1973	104396			
			1.29 (1.19–1.40)	36.71	1.37×10^-9^
rs10922273-TT & ever smoked	25	1368			
rs10922273-CC & never smoked	2014	107214			
			0.97 (0.65–1.45)	0.001	0.97
rs10922273-CT & ever smoked	572	29845			
rs10922273-CC & never smoked	2014	107214			
			1.02 (0.93–1.12)	0.16	0.96
rs10033900-TT & ever smoked	1101	44817			
rs10033900-CC & never smoked	667	34843			
			1.28 (1.16–1.41)	25.19	5.20×10^-7^
rs10033900-CT & ever smoked	2068	97620			
rs10033900-CC & never smoked	667	34843			
			1.11 (1.01–1.21)	4.98	0.03

ORs with 95% CIs were calculated using the Wald normal approximation method. Pearson’s chi-squared test was employed to assess associations.

In the CFI locus, the risk variant rs10033900-T showed significant positive additive (RERI=0.11; 95% CI: 0.04–0.17, AP=0.10; 95% CI: 0.03–0.16) and multiplicative interactions (HR_mi_=1.11; 95% CI: 1.03–1.19; p=5.20×10^-3^) with smoking status. Homozygous rs10033900-T carriers who had ever smoked (OR=1.28, p=5.20×10^-7^) and heterozygous carriers who had ever smoked (OR=1.11, p=0.03) also exhibited a significantly elevated risk of AMD compared to rs10033900-CC carriers who had never smoked.

### Distinct complement protein profiles across AMD PRS and smoking status groups

The expression levels of 28 complement pathway proteins were subsequently analyzed across six compared groups to identify potential DE patterns (Supplementary file: Table S5 and Figure 7, for detailed protein level results, along with information on the significance of differential expression).

Following adjustment for age, sex, BMI, alcohol consumption status, vascular/heart diseases, diabetes, medications for cholesterol, blood pressure, or diabetes, and exogenous hormone use, CD55, CFHR4, and CFHR5 were found to exhibit significant associations with AMD. Furthermore, logistic regression analyses demonstrated that fourteen complement pathway proteins, including SERPING1, CFB, CFI, CFHR5, C1S, FCN2, FCN1, MBL2, C3, CR2, CLU, CR1, C4BPB and CFP, were significantly associated with high PRS participants compared to those with low PRS. The association between CFHR5 and AMD was further validated through both MR and colocalization analyses. MR results additionally implicated CFHR4, CFD, and MBL2 as causal proteins for AMD (Supplementary file: Table S6 and Figure 8A), while colocalization analysis identified C3, CD46, CFD, CFI, and MASP1 as potential contributors to the pathogenesis of AMD (Supplementary file: Table S7 and Figure 8B).

In the analysis of smoking status, significant differences were observed in eleven proteins, including CFHR2, CFHR5, CFHR4, C2, CD46, C4BPB, C3, C7, CFH, CFB and CFP, with eight of these remaining significant after correction for multiple testing. Notably, CFHR2 exhibited a strong association with smoking (OR=2.16, p<2×10^-16^), underscoring a robust relationship between smoking and dysregulation of complement proteins, independent of genetic predisposition. The association between CFB and ever smoked was further validated through MR analysis. Additionally, MR analysis identified CFD, C1RL, and MBL2 as other complement proteins associated with ever smoked.

Among participants with low PRS and ever smoked exhibited elevated levels of SERPING1, CFI, CFHR5, CFB, C5, C1S, CFH, FCN2, FCN1, and MBL2, alongside decreased levels of CR2, CR1, C4BPB and CFP compared to individuals who have low PRS and never smoked (p<0.05). In individuals with high PRS who had never smoked, only eight proteins (CFHR2, CFHR5, CFHR4, C4BPB, CFH, CFB, CFP, C2) were significantly dysregulated (p<0.05) relative to individuals who have low PRS and never smoked. Participants with both high PRS and a history of smoking exhibited the most pronounced differential expression (18 proteins: CFHR2, SERPING1, CFHR5, CFHR4, C2, CFI, C1S, FCN2, CD46, FCN1, CR2, CLU, C4BPB, CFB, C3, CFH, CR1, CFP), suggesting the synergistic effects of genetic predisposition to AMD and smoking history in modulating complement protein expression.

## DISCUSSION

Our meta-GWAS analysis identified two novel AMD risk loci: OCA2 and NOA1. OCA2 is notable for its role in eye pigmentation^26^ and its established association with the eye color phenotype^27^. The newly identified NOA1 locus encodes a protein that regulates mitochondrial respiratory complexes in an oxygen-dependent manner, playing a critical role in oxidative stress and apoptosis, which are key processes in AMD pathogenesis^28,29^. We constructed a PRS based on the meta-AMD GWAS using the PRS-CS model. Compared to the null model (AUC=0.74), which included age, sex, smoking status, and the top 10 principal components, the AUC increased to 0.755 when incorporating the AMD PRS, a statistically significant improvement (p=2×10^-16^). Although the UKBB dataset includes the standard PRS developed by Thompson et al.^30^ (UKBB Data-Field 26204), the corresponding discovery AMD GWAS datasets were not made available. To conduct variant–smoking interaction analyses, which require causal variants from the discovery GWAS, we recalculated the PRS using a recently released meta-analysis of four independent, large-scale AMD GWAS datasets with no sample overlap. This recalculated PRS was used in subsequent analyses and showed strong predictive accuracy. In the UKBB cohort, AMD prevalence was approximately 2.0%, increasing to 3.3% in individuals with a high PRS and dropping to 1.4% in those with a low PRS.

Smoking is a well-established modifiable risk factor for AMD, supported by extensive epidemiological evidence. However, its interaction with genetic susceptibility in influencing AMD onset and progression has been an area of active research. In addition to smoking status, female sex, age, diabetes, body mass index, and education level (O levels/GCSEs or equivalent qualifications) were also identified as potential positive risk factors for AMD. In this study, we provide the first large-scale, population-based prospective analysis confirming that both the attributable risks (additive) and the differences in smoking-related hazard ratios (multiplicative) increased from low to high genetic risk groups. Previous studies have also highlighted gene-environment interactions in AMD. For example, Schmidt et al.^31^ found that the APOE genotype influences the smoking-related risk of AMD, with a more pronounced effect on the development of choroidal neovascularization (CNV). Notably, smoking posed the greatest risk to individuals carrying the apolipoprotein ε2 allele. Likewise, Baird et al.^32^ found that the Y402H variant in the CFH gene not only drives disease progression but also interacts with smoking and pathogen exposure. Our variant-level analysis revealed synergistic interactions between ever smoked and three CFH locus variants: rs4388642-C, rs10922273-T, and rs17562659-C. Although these variants differ from those previously reported, our results indicate a broad interaction between smoking and CFH polymorphisms. Additionally, we identified a novel positive interaction between smoking and the CFI locus, particularly with the rs10033900-T variant. The rs10033900 variant within the CFI locus has been consistently associated with AMD risk across multiple studies, highlighting its potential role in AMD susceptibility^6,33^. Previous research has also explored the interaction between the CFI locus, smoking, and AMD. For example, Seddon et al.^34^ reported that type 1 CFI carriers, identified by 23 rare variants associated with low serum factor I (FI) levels and reduced FI function, exhibited a positive association with progression to geographic atrophy (GA) among individuals who never smoked (OR=2.4; 95% CI: 0.9–6.0; p=0.07). However, this study found no significant interaction between smoking and CFI carrier status with respect to the progression of either GA or neovascular AMD (NV).

In participants with AMD or a high PRS, we observed marked dysregulation of CFHR4 and CFHR5, reinforcing the hypothesis that activation of the alternative complement pathway, mediated by dysregulation of the CFHR protein family^35^, plays a crucial role in the pathogenesis of AMD. Furthermore, our findings highlighted significant alterations in the expression of complement proteins, including SERPING1, CFHR5, FCN1, and FCN2, between individuals with a high PRS and those with a low PRS. SERPING1, a key regulator of the classical complement pathway, modulates complement activation by inhibiting C1 esterase activity^36^. Additionally, FCN1 and FCN2, central components of the lectin pathway, initiate complement activation by recognizing pathogen- or damage-associated molecular patterns (PAMPs or DAMPs). The widespread activation of the complement system, driven by the combined effects of the classical and lectin pathways, likely exacerbates inflammation and tissue damage, accelerating AMD progression. In individuals who have ever smoked and high PRS, we identified 18 distinct complement proteins, including CFHR2, CFHR5, SERPING1, CFI, C2, FCN2, and FCN1, underscoring the synergistic effects of genetic predisposition to AMD and smoking history. It is well-established that smoking contributes to oxidative stress, inflammation, and endothelial dysfunction, which serve as potent activators of the complement system, particularly the alternative, classical, and lectin pathways. This activation can further disrupt the regulation of complement proteins, thereby exacerbating inflammation and retinal tissue damage. In individuals with elevated PRS, smoking may potentiate these effects, perpetuating a detrimental cycle of complement-mediated damage that accelerates the progression of AMD. Targeting the complement system, particularly through inhibition of key proteins like CFHR2, and CFHR5, could offer a promising therapeutic strategy. Complement inhibitors such as pegcetacoplan and avacincaptad pegol, which are currently being evaluated in clinical trials, hold potential for slowing AMD progression, particularly in individuals with high genetic risk and ever smoked. Additionally, anti-inflammatory therapies aimed at reducing oxidative stress or modulating the lectin and classical complement pathways could provide new avenues for treatment, offering hope for personalized therapies tailored to both genetic makeup and lifestyle factors.

### Strengths and limitations

This study highlights key implications for public health and clinical practice. First, targeted smoking cessation campaigns are crucial, especially for individuals with high genetic risk, emphasizing the combined impact of smoking and genetic susceptibility on AMD risk. Second, integrating genetic risk assessments into AMD prevention programs, such as genetic testing for those with a family history, can enable personalized risk stratification and early interventions like regular eye examinations and advanced imaging. Clinicians should offer personalized counseling, stressing modifiable risk factors like smoking and lifestyle changes.

Despite the strengths of our large-scale cohort analysis, several limitations should be noted. First, the observational design and potential residual confounding preclude definitive causal conclusions. Second, the predominantly European-ancestry cohort limits generalizability to other populations. Third, inherent weaknesses of MR, particularly regarding unobserved pleiotropy, may also bias causal estimates. Future studies with individual-level data or multivariable approaches could further unravel whether complement proteins mediate risk independently of other pathways. Fourth, although the PRS was computed, its predictive accuracy may vary across cohorts, and our focus on complement-related loci might overlook other AMD-relevant pathways. Finally, interaction models in our study were restricted to linear models, and did not investigate potential non-linear relationships and sex-specific differences. Future studies integrating machine learning and sex-specific methods could address these gaps and dissect complex non-linear interactions.

## CONCLUSIONS

Our analysis uncovered two novel risk loci for AMD, OCA2 and NOA1, expanding the genetic understanding of the disease. The PRS significantly enhanced AMD risk prediction, with a notable increase in the AUC in 331281 UK Biobank participants. Long-term follow-up data revealed significant interactions between smoking history and PRS, particularly at the CFH and CFI loci. Notably, we observed significant dysregulation of complement proteins, including CFHR4, CFHR5, CFB, and C3, which were strongly associated with both high PRS and smoking history, suggesting the synergistic effects of genetic predisposition and smoking in modulating complement protein expression. Our findings underscore the importance of both genetic and environmental factors, particularly smoking, in influencing complement pathway activation and contributing to AMD pathology.

## Supplementary Material



## Data Availability

The download links for the original GWAS data, along with details on data cleaning, processing, and analysis, are provided in the Supplementary file Methods. The data supporting this research can be found in the Supplementary file.
