# Supplementary material for: Deciphering gene–smoking interactions in age-related macular degeneration through cross-biobank genomic integration
Source: Tob Induc Dis. 2025 Jul 29;23:10.18332/tid/205419. doi: 10.18332/tid/205419 (PMC12306451; doi:10.18332/tid/205419)
Supplement: Supplementary file 1 [file TID-23-108-s1.pdf]

Table S1.Complement Pathway Protein Names Targeted in This Study

Protein names	Gene names	UniProt ID
Complement C1q Binding Protein	C1QBP	Q07021
Complement C1r Subcomponent Like	C1RL	Q9NZP8
Complement C1r	C1R	P00736
Complement C1s	C1S	P09871
Complement C2	C2	P06681
Complement C3	C3	P01024
Complement Component 4 Binding Protein Beta	C4BPB	P20851
Complement C5	C5	P01031
Complement C7	C7	P10643
Complement C9	C9	P02748
CD46 Molecule	CD46	P15529
CD55 Molecule (Cromer Blood Group)	CD55	P08174
Complement Factor B	CFB	P00751
Complement Factor D	CFD	P00746
Complement Factor H Related 2	CFHR2	P36980
Complement Factor H Related 4	CFHR4	Q92496
Complement Factor H Related 5	CFHR5	Q9BXR6
Complement Factor H	CFH	P08603
Complement Factor I	CFI	P05156
Complement Factor Properdin	CFP	P27918
Clusterin	CLU	P10909
Complement C3b/C4b Receptor 1 (Knops Blood Group)	CR1	P17927
Complement C3d Receptor 2	CR2	P20023
Ficolin 1	FCN1	O00602
Ficolin 2	FCN2	Q15485
MBL Associated Serine Protease 1	MASP1	P48740
Mannose Binding Lectin 2	MBL2	P11226
Serpin Family G Member 1	SERPING1	P05155

Table S2. Identification of 79 Independent Lead AMD Risk Variants in 43 Loci

Serial number	Locus name	IND_LEAD_SNP	LOCI_START
1	CFH	rs6428282	194767072
		rs1538596	194767072
		rs1451924	194767072
		rs10754176	194767072
		rs6683628	194767072
		rs994454	194767072
		rs12727325	194767072
		rs16840224	194767072
		rs6677604	194767072
		rs3753396	194767072
		rs4915318	194767072
		rs1855115	194767072
		rs12402465	194767072
		rs517222	194767072
		rs1337005	194767072
		rs16842154	194767072
		rs1125952	194767072
		rs12046946	194767072
2	PTPRC	rs12732681	197933606
		rs10494783	197933606
3	CD55	rs11120438	206886597
4	CD46	rs2724374	207441191
5	PLEKHM3	rs2718666	208296905
6	COL4A3	rs11884770	227586920
7	ADAMTS9-AS	rs13096392	64222611
8	COL8A1	rs2315840	99329320
9	ZBTB38	rs6785073	140639330
10	NOA1	rs1713998	57344902
11	AFF1	rs3775220	87514753
12	CFI	rs10033900	110159067
13	SPEF2	rs1508662	35067236
14	TMEM161B	rs2194026	87301273
15	ADAM19	rs6899205	156443285
16	C2/CFB/SKIV2L	rs3131635	30976134
		rs4151671	30976134
		rs522162	30976134
		rs204896	30976134
		rs28366298	30976134
		rs2857107	30976134
17	VEGFA	rs943080	43326627
18	COL10A1	rs1064583	115946576
19	KMT2E/SRPK2	rs10808141	104215490
20	RORB	rs10781177	76093011
21	TGFBR1	rs7874221	101369068
22	ARMS2/HTRA1	rs12571870	123271303
		rs2245770	123271303
		rs11200430	123271303
		rs2281670	123271303
		rs12262185	123271303

		rs4751881	123271303
		rs984668	123271303
		rs2248799	123271303
		rs2249049	123271303
		rs763720	123271303
23	RDH5/CD63	rs3138136	55617570
24	B3GALTL	rs4943289	31335375
25	RAD51B	rs2208397	68253287
		rs2842343	68253287
26	DPF3	rs2110552	72855131
27	OCA2	rs12913832	27865618
28	TRPM1	rs7182946	30894868
29	LIPC	rs7350789	58179668
		rs1077835	58179668
30	SMAD3	rs6494627	66838914
		rs17293632	66838914
31	CSK	rs1378942	74577367
32	RLBP1	rs3825991	89261664
34	CETP	rs1800775	56495236
35	CTRB2/CTRB1	rs8048816	74922201
36	CLUL1	rs9973159	97950
37	FUT6	rs8111600	5321968
38	C3	rs2250656	6218534
		rs339392	6218534
		rs171094	6218534
39	ANKLE1	rs8170	16889704
40	APOE	rs4420638	44922946
41	C20orf85	rs8125204	56132192
42	SYN3/TIMP3	rs5754222	32603968
43	PDGFB	rs879180	39131547

LOCI_END	CHR	BP	EA	NEA
198571724	1	195267072	A	C
198571724	1	195459410	A	C
198571724	1	195909031	A	G
198571724	1	196115914	T	C
198571724	1	196177193	A	G
198571724	1	196209649	A	G
198571724	1	196223735	A	G
198571724	1	196569681	T	C
198571724	1	196686918	A	G
198571724	1	196695742	A	G
198571724	1	196897088	A	C
198571724	1	197046782	A	C
198571724	1	197167465	T	C
198571724	1	197227033	A	G
198571724	1	197265641	T	C
198571724	1	197910646	T	C
198571724	1	197968474	A	G
198571724	1	198071724	A	C
199163661	1	198433606	A	G
199163661	1	198663661	A	G
207886597	1	207386597	A	G
208441191	1	207941191	T	G
209296905	2	208796905	T	C
228586920	2	228086920	T	C
65222611	3	64722611	T	G
100329320	3	99829320	A	C
141639330	3	141139330	A	G
58344902	4	57844902	A	G
88514753	4	88014753	A	G
111159067	4	110659067	T	C
36067236	5	35567236	T	C
88301273	5	87801273	T	G
157443285	5	156943285	A	G
33285515	6	31476134	A	G
33285515	6	31918903	T	C
33285515	6	31919917	T	C
33285515	6	32064098	T	C
33285515	6	32560859	A	C
33285515	6	32785515	T	C
44326627	6	43826627	T	C
116946576	6	116446576	A	G
105215490	7	104715490	T	G
77093011	9	76593011	T	C
102369068	9	101869068	T	C
124726215	10	123771303	A	C
124726215	10	123782002	T	C
124726215	10	123904707	A	G
124726215	10	123976029	A	G
124726215	10	124014939	A	G

124726215	10	124015629	T	C
124726215	10	124120559	A	G
124726215	10	124223944	T	C
124726215	10	124226215	A	G
124726215	10	124262444	A	G
56617570	12	56117570	T	C
32335375	13	31835375	A	G
69477490	14	68753287	T	G
69477490	14	68977490	A	G
73855131	14	73355131	T	C
28865618	15	28365618	A	G
31894868	15	31394868	T	G
59223426	15	58679668	A	G
59223426	15	58723426	A	G
67942596	15	67338914	A	G
67942596	15	67442596	T	C
75577367	15	75077367	A	C
90261664	15	89761664	A	C
57495236	16	56995236	A	C
75922201	16	75422201	A	C
1097950	18	597950	T	C
6321968	19	5821968	T	C
7227365	19	6718534	T	C
7227365	19	6722022	T	G
7227365	19	6727365	A	G
17889704	19	17389704	A	G
45922946	19	45422946	A	G
57132192	20	56632192	T	C
33603968	22	33103968	T	C
40131547	22	39631547	T	C

EAF	BETA	SE	P	COJO_BETA
0.54	6.07E-03	1.45E-03	2.67E-05	8.53E-03
0.24	-1.10E-02	1.68E-03	6.08E-11	-9.99E-03
0.70	-6.76E-03	1.57E-03	1.77E-05	-9.10E-03
0.50	1.59E-03	1.44E-03	2.71E-01	-8.37E-03
0.55	6.34E-03	1.48E-03	1.70E-05	1.02E-02
0.91	-1.38E-02	2.54E-03	5.35E-08	-1.55E-02
0.27	4.28E-02	1.63E-03	1.43E-151	1.47E-02
0.33	-5.46E-02	1.53E-03	5.60E-280	-3.67E-02
0.20	-5.43E-02	1.79E-03	3.08E-202	-7.27E-02
0.80	1.01E-02	1.81E-03	2.32E-08	3.92E-02
0.29	-5.99E-02	1.58E-03	1.67E-308	-5.38E-02
0.90	3.76E-02	2.37E-03	1.12E-56	3.37E-02
0.26	1.23E-02	1.64E-03	5.16E-14	1.65E-02
0.21	-4.38E-03	1.76E-03	1.25E-02	-3.86E-02
0.50	-2.26E-02	1.44E-03	1.18E-55	-2.74E-02
0.89	-1.54E-04	2.30E-03	9.46E-01	-1.64E-02
0.58	1.40E-02	1.46E-03	1.09E-21	1.14E-02
0.21	-1.43E-02	1.75E-03	3.28E-16	-1.21E-02
0.58	6.60E-03	1.46E-03	6.26E-06	1.34E-02
0.11	-2.02E-02	2.28E-03	7.10E-19	-1.95E-02
0.55	8.98E-03	1.45E-03	5.91E-10	8.86E-03
0.74	1.16E-02	1.65E-03	1.57E-12	1.14E-02
0.44	8.41E-03	1.45E-03	6.69E-09	8.41E-03
0.33	-1.01E-02	1.53E-03	3.78E-11	-1.01E-02
0.59	-1.08E-02	1.47E-03	1.46E-13	-1.08E-02
0.08	1.86E-02	2.60E-03	8.22E-13	1.86E-02
0.36	8.46E-03	1.50E-03	1.81E-08	8.46E-03
0.65	-8.61E-03	1.51E-03	1.24E-08	-8.61E-03
0.59	-1.25E-02	1.46E-03	1.59E-17	-1.25E-02
0.51	1.10E-02	1.44E-03	2.81E-14	1.10E-02
0.90	1.49E-02	2.41E-03	7.59E-10	1.49E-02
0.17	1.20E-02	1.92E-03	3.84E-10	1.20E-02
0.31	-8.82E-03	1.56E-03	1.41E-08	-8.82E-03
0.32	-1.80E-03	1.55E-03	2.45E-01	-9.66E-03
0.10	-3.47E-02	2.37E-03	2.17E-48	-4.23E-02
0.87	4.81E-02	2.15E-03	1.17E-110	5.51E-02
0.13	-2.63E-02	2.13E-03	6.66E-35	-1.46E-02
0.71	-9.39E-03	1.58E-03	3.13E-09	-1.31E-02
0.13	4.65E-03	2.11E-03	2.76E-02	1.58E-02
0.53	1.57E-02	1.44E-03	2.18E-27	1.57E-02
0.61	9.89E-03	1.48E-03	2.13E-11	9.89E-03
0.64	-1.14E-02	1.50E-03	3.98E-14	-1.14E-02
0.46	-8.26E-03	1.44E-03	1.08E-08	-8.26E-03
0.79	1.12E-02	1.77E-03	2.44E-10	1.12E-02
0.44	8.36E-03	1.45E-03	8.68E-09	9.36E-03
0.43	-8.13E-03	1.46E-03	2.41E-08	-1.11E-02
0.09	9.83E-03	2.53E-03	1.05E-04	2.10E-02
0.81	-1.66E-02	1.85E-03	2.72E-19	-1.30E-02
0.37	-1.37E-02	1.50E-03	5.89E-20	-1.60E-02

0.09	-7.08E-03	2.48E-03	4.23E-03	-2.27E-02
0.40	-6.86E-03	1.47E-03	3.09E-06	-1.50E-02
0.48	5.51E-02	1.44E-03	1.67E-308	1.12E-01
0.74	3.53E-02	1.63E-03	1.50E-103	1.18E-01
0.22	4.43E-02	1.73E-03	1.05E-144	1.35E-02
0.13	1.33E-02	2.15E-03	5.68E-10	1.33E-02
0.33	-8.72E-03	1.54E-03	1.41E-08	-8.72E-03
0.32	-1.23E-02	1.54E-03	1.77E-15	-1.40E-02
0.16	1.11E-02	1.99E-03	2.72E-08	1.38E-02
0.64	8.84E-03	1.51E-03	4.25E-09	9.09E-03
0.21	-1.09E-02	1.76E-03	6.16E-10	-1.13E-02
0.55	7.82E-03	1.45E-03	6.83E-08	8.16E-03
0.38	-1.20E-02	1.49E-03	7.85E-16	-1.22E-02
0.73	1.16E-02	1.63E-03	1.15E-12	1.20E-02
0.47	-7.53E-03	1.44E-03	1.79E-07	-8.29E-03
0.28	-8.09E-03	1.61E-03	4.70E-07	-9.56E-03
0.60	1.08E-02	1.47E-03	1.86E-13	1.13E-02
0.48	1.31E-02	1.44E-03	9.23E-20	1.31E-02
0.49	1.36E-02	1.44E-03	3.02E-21	1.36E-02
0.73	9.34E-03	1.62E-03	9.00E-09	9.34E-03
0.18	-1.14E-02	1.86E-03	8.34E-10	-1.14E-02
0.09	-1.63E-02	2.53E-03	1.31E-10	-1.56E-02
0.68	1.40E-02	1.54E-03	1.21E-19	1.23E-02
0.77	6.08E-03	1.72E-03	4.22E-04	2.28E-02
0.66	-1.27E-02	1.52E-03	6.05E-17	-1.90E-02
0.21	1.23E-02	1.84E-03	2.02E-11	1.23E-02
0.74	1.49E-02	1.65E-03	1.63E-19	1.49E-02
0.15	-1.14E-02	2.00E-03	1.25E-08	-1.14E-02
0.82	1.51E-02	1.89E-03	1.37E-15	1.53E-02
0.29	-9.92E-03	1.59E-03	4.92E-10	-1.01E-02

COJO_SE	COJO_P	Overlapped with published AMD GWAS lead loci
1.45E-03	4.10E-09	
1.68E-03	2.97E-09	
1.58E-03	8.60E-09	
1.48E-03	1.49E-08	
1.51E-03	1.24E-11	
2.61E-03	2.88E-09	
1.78E-03	1.57E-16	
2.02E-03	6.41E-74	
2.21E-03	3.34E-237	37634759
2.14E-03	9.29E-75	
2.37E-03	8.15E-114	
2.90E-03	2.91E-31	
2.08E-03	2.43E-15	
2.12E-03	3.61E-74	
2.33E-03	7.23E-32	
2.35E-03	2.67E-12	
1.51E-03	3.94E-14	
1.80E-03	1.88E-11	26691988
1.51E-03	8.70E-19	
2.30E-03	2.44E-17	
1.45E-03	1.01E-09	37634759
1.65E-03	4.91E-12	32843070
1.45E-03	6.71E-09	37634759
1.53E-03	3.77E-11	26691988
1.47E-03	1.46E-13	26691988
2.60E-03	8.26E-13	26691988
1.50E-03	1.80E-08	37634759
1.51E-03	1.24E-08	novel
1.46E-03	1.59E-17	37634759
1.44E-03	2.81E-14	26691988
2.41E-03	7.61E-10	37634759
1.92E-03	3.83E-10	FinnGen
1.56E-03	1.41E-08	37634759
1.58E-03	1.03E-09	26691988
2.40E-03	1.87E-69	
2.39E-03	2.58E-117	
2.27E-03	1.44E-10	
1.68E-03	5.68E-15	
2.31E-03	8.02E-12	
1.44E-03	2.19E-27	26691988
1.48E-03	2.13E-11	37634759
1.50E-03	3.98E-14	26691988
1.44E-03	1.08E-08	37634759
1.77E-03	2.44E-10	37634759
1.48E-03	2.31E-10	26691988
1.48E-03	8.40E-14	
2.59E-03	5.73E-16	
2.09E-03	5.11E-10	
1.56E-03	9.09E-25	

2.78E-03	3.13E-16	
1.50E-03	1.42E-23	
1.89E-03	0.00E+00	
2.16E-03	0.00E+00	
1.82E-03	1.47E-13	
2.15E-03	5.69E-10	26691988
1.54E-03	1.41E-08	37634759
1.56E-03	2.90E-19	37634759
2.01E-03	6.08E-12	37634759
1.51E-03	1.58E-09	FinnGEN
1.77E-03	1.69E-10	novel
1.45E-03	1.85E-08	37634759
1.49E-03	1.97E-16	26691988
1.63E-03	1.61E-13	
1.45E-03	1.06E-08	37634759
1.61E-03	3.04E-09	
1.47E-03	1.35E-14	37634759
1.44E-03	9.23E-20	37634759
1.44E-03	3.03E-21	26691988
1.62E-03	8.98E-09	26691988
1.86E-03	8.36E-10	37634759
2.53E-03	7.49E-10	37634759
1.71E-03	6.06E-13	26691988
2.11E-03	3.91E-27	
1.95E-03	1.80E-22	
1.84E-03	2.02E-11	FinnGEN
1.65E-03	1.63E-19	37634759
2.00E-03	1.25E-08	26691988
1.89E-03	5.74E-16	37634759
1.59E-03	2.04E-10	37634759

Table S3 Replication of Causal Variants Identified by Three Finemaping Methods in UKBB D

Locus name	SNP	CHR	BP	UKB_EA	UKB_NEA	UKB_EAF
CFH	rs6677435	1	195790658	A	G	0.04
CFH	rs4388642	1	196494547	C	T	0.31
CFH	rs1756265	1	197450636	C	T	0.10
CFH	rs1049475	1	197507411	T	C	0.07
CFH	rs1092227	1	197725373	T	C	0.09
CFH	rs1125952	1	197968474	A	G	0.64
PTPRC	rs1049478	1	198663661	A	G	0.05
NOA1	rs1713998	4	57844902	G	A	0.37
CFI	rs1003390	4	110659067	C	T	0.52
C2/CFB/SKIV2L	rs9273440	6	32627561	C	T	0.74
VEGFA	rs943080	6	43826627	T	C	0.50
COL10A1	rs1064583	6	116446576	G	A	0.40
CFI	rs3138136	12	56117570	T	C	0.11
OCA2	rs1291383	15	28365618	G	A	0.79
RLBP1	rs3825991	15	89761664	A	C	0.47
CETP	rs1800775	16	56995236	A	C	0.49
CLUL1	rs9973159	18	597950	T	C	0.15
FUT6	rs8111600	19	5821968	T	C	0.04
C3	rs2250656	19	6718534	C	T	0.29
APOE	rs4420638	19	45422946	G	A	0.19
SYN3/TIMP3	rs5754222	22	33103968	C	T	0.13

Datasets

UKB_BETA	UKB_SE	UKB_P	Meta_EA	Meta_NEA	Meta_EAF	Meta_BETA	Meta_SE
-0.09	0.05	4.07E-02	A	G	0.11	-0.02	2.29E-03
0.16	0.02	1.82E-17	T	C	0.64	-0.06	1.50E-03
0.12	0.03	3.78E-05	T	C	0.86	-0.03	2.08E-03
0.15	0.03	2.78E-05	T	C	0.12	0.03	2. 19E-03
-0.06	0.03	4.62E-02	T	C	0.18	-0.02	1.86E-03
0.00	0.02	8.08E-01	A	G	0.58	0.01	1.46E-03
0.00	0.04	9. 13E-01	A	G	0.11	-0.02	2.28E-03
0.02	0.02	2.99E-01	A	G	0.65	-0.01	1.51E-03
-0.07	0.02	4.92E-05	T	C	0.51	0.01	1.44E-03
0.05	0.02	2.30E-02	T	C	0.26	-0.02	1.69E-03
0.03	0.02	8. 10E-02	T	C	0.53	0.02	1.44E-03
-0.03	0.02	6.67E-02	A	G	0.61	0.01	1.48E-03
0.05	0.03	9.33E-02	T	C	0.13	0.01	2. 15E-03
0.00	0.02	8.67E-01	A	G	0.21	-0.01	1.76E-03
0.04	0.02	4.84E-02	A	C	0.48	0.01	1.44E-03
0.08	0.02	3.35E-06	A	C	0.49	0.01	1.44E-03
-0.05	0.03	6.96E-02	T	C	0.18	-0.01	1.86E-03
-0.22	0.05	6.68E-06	T	C	0.09	-0.02	2.53E-03
-0.07	0.02	7.09E-04	T	C	0.68	0.01	1.54E-03
-0.12	0.02	8.79E-07	A	G	0.74	0.01	1.65E-03
-0.09	0.03	5.44E-04	T	C	0.82	0.02	1.89E-03

Meta_P	PP_FINEMAP	PP_ABF	PP_SUSIE
3.68E-13	0.96	0.96	0.95
7.16E-297	1.00	1.00	1.00
7.22E-43	0.90	0.90	0.91
7.73E-42	1.00	1.00	1.00
2.63E-28	0.87	0.90	0.90
1.09E-21	0.99	0.99	0.99
7.10E-19	1.00	1.00	1.00
1.24E-08	0.96	0.96	0.95
2.81E-14	0.95	0.94	0.94
7.38E-28	1.00	1.00	1.00
2.18E-27	1.00	1.00	1.00
2.13E-11	0.82	0.83	0.82
5.68E-10	0.99	0.99	0.99
6.16E-10	0.98	0.98	0.98
9.23E-20	0.95	0.95	0.95
3.02E-21	1.00	1.00	1.00
8.34E-10	0.87	0.88	0.87
1.31E-10	1.00	1.00	1.00
1.21E-19	1.00	1.00	1.00
1.63E-19	1.00	1.00	1.00
1.37E-15	1.00	1.00	1.00

Table S4.Summary of Results for Variant-Smoking Interactions

Variant	Recode			HR_smoke	CI5_smoke
rs6677435-A	AA (511)	AG(25016)	GG(303457)	1.10	1.04
rs4388642-C	CC(31129)	CT(140636)	TT(159516)	1.04	0.96
rs17562659-C	CC(3081)	CT(58256)	TT(267415)	1.06	1.01
rs10494757_T	CC(287953)	CT(39878)	TT(1342)	1.08	1.03
rs10922273-T	TT(2291)	TC(50655)	CC(275069)	1.13	1.07
rs1125952-G	GG(42397)	GA(151777)	AA(135928)	1.14	1.06
rs10494783-A	AA(839)	AG(31767)	GG(298116)	1.09	1.04
rs1713998_G	AA(131913)	AG(152006)	GG(43563)	1.12	1.04
rs10033900-T	TT(76313)	TC(165447)	CC(89521)	0.99	0.91
rs9273440_C	TT(18795)	TC(114255)	CC(169031)	1.06	0.92
rs943080-T	TT(81312)	TC(164882)	CC(83564)	1.11	1.01
rs1064583-G	GG(52409)	GA(158478)	AA(120394)	1.11	1.03
rs3138136-T	TT(4318)	TC(66713)	CC(260250)	1.09	1.03
rs12913832-A	AA(15292)	AG(111473)	GG(204516)	1.13	1.06
rs3825991_A	CC(88209)	CA(160144)	AA(72025)	1.06	0.97
rs1800775-A	AA(78208)	AC(165498)	CC(87575)	1.15	1.05
rs9973159-T	TT(7148)	TC(83103)	CC(241030)	1.10	1.04
rs8111600-T	TT(494)	TC(25521)	CC(303972)	1.10	1.04
rs2250656_C	TT(157396)	TC(122212)	CC(23256)	1.06	0.99
rs4420638-G	GG(12118)	GA(102368)	AA(216795)	1.07	1.01
rs5754222-C	CC(6107)	CT(76978)	TT(247938)	1.10	1.04

CI95_smoke	*P* value_smoke	HR_variant	CI5_variant	CI95_variant	*P* value_variant
1.16	3.29E-04	0.98	0.85	1.13	7.62E-01
1.11	3.40E-01	1.12	1.05	1.19	2.03E-04
1.13	2.81E-02	1.04	0.95	1.15	3.60E-01
1.15	3.19E-03	1.10	0.98	1.22	1.01E-01
1.19	1.66E-05	1.08	0.98	1.19	1.08E-01
1.23	3.37E-04	1.03	0.97	1.09	3.18E-01
1.15	8.28E-04	0.99	0.87	1.12	8.37E-01
1.20	3.62E-03	1.04	0.98	1.10	2.22E-01
1.08	8.15E-01	1.01	0.95	1.06	8.60E-01
1.22	3.99E-01	1.04	0.97	1.11	2.75E-01
1.21	2.20E-02	1.04	0.98	1.09	2.25E-01
1.19	8.01E-03	0.98	0.92	1.04	4.64E-01
1.15	3.66E-03	1.04	0.95	1.13	4.21E-01
1.21	8.97E-05	1.05	0.98	1.13	1.33E-01
1.15	1.88E-01	1.02	0.96	1.08	5.19E-01
1.25	2.11E-03	1.11	1.05	1.18	1.60E-04
1.17	9.95E-04	0.97	0.89	1.05	4.19E-01
1.16	4.96E-04	0.85	0.73	0.99	3.52E-02
1.13	1.21E-01	0.90	0.84	0.96	1.86E-03
1.13	2.81E-02	0.86	0.80	0.93	7.43E-05
1.16	1.65E-03	0.92	0.85	1.00	6.01E-02

RERI	AP	SI
-0. 129[-0.31-0.0527]	-0. 135[-0.334-0.0635]	-0.634[NaN-NaN]
0. 1[0.0236-0. 177]	0.0801[0.0175-0. 143]	1.65[0.926-2.94]
0. 126[0.000794-0.25]	0. 102[0.00279-0.2]	2. 15[0.724-6.38]
0.0832[-0.0726-0.239]	0.0658[-0.0551-0. 187]	1.46[0.657-3.24]
-0.226[-0.362--0.09]	-0.229[-0.375--0.0842]	-0.0686[NaN-NaN]
-0.0645[-0.144-0.0152]	-0.0582[-0.129-0.0123]	0.627[0.406-0.968]
-0.0159[-0.181-0. 149]	-0.0149[-0.17-0. 14]	0.804[0.0892-7.25]
-0.0285[-0.106-0.0493]	-0.0254[-0.094-0.0432]	0.814[0.505-1.31]
0. 105[0.0391-0. 171]	0.0954[0.0317-0. 159]	-19.5[NaN-NaN]
0.0168[-0.0678-0. 101]	0.0151[-0.0621-0.0922]	1. 17[0.431-3. 16]
-0.011[-0.0848-0.0628]	-0.00975[-0.0746-0.0551]	0.922[0.564-1.51]
-0.0207[-0.0959-0.0545]	-0.0194[-0.0894-0.0506]	0.76[0.345-1.68]
0.0257[-0.0904-0. 142]	0.0223[-0.0783-0. 123]	1.21[0.483-3.02]
-0.0891[-0.182-0.00342]	-0.0813[-0.165-0.0029]	0.52[0.287-0.941]
0.0295[-0.0425-0. 102]	0.0267[-0.0394-0.0928]	1.38[0.458-4. 15]
-0.0428[-0.119-0.0332]	-0.0352[-0.0963-0.0259]	0.836[0.644-1.08]
-0.035[-0.137-0.0668]	-0.0338[-0.132-0.0648]	0.5[0.0753-3.32]
-0.0721[-0.243-0.0988]	-0.0826[-0.283-0. 118]	2.3[0.148-35.8]
0.0521[-0.0287-0. 133]	0.0517[-0.0293-0. 133]	-0. 161[NaN-NaN]
0.05[-0.0374-0. 137]	0.051[-0.0382-0. 14]	0.297[0.0237-3.73]
-0.0175[-0.121-0.0861]	-0.0175[-0.121-0.0863]	0.0417[1.35e-42-1.29e+39]

Multiplicative interaction	*P*
0.883[0.733-1.06]	1.86E-01
1.08[1.01-1.17]	3.55E-02
1.11[0.989-1.25]	7.55E-02
1.06[0.927-1.22]	3.83E-01
0.806[0.71-0.916]	9.12E-04
0.942[0.875-1.01]	1.08E-01
0.986[0.839-1.16]	8.68E-01
0.972[0.903-1.05]	4.41E-01
1.11[1.03-1.19]	5.20E-03
1.01[0.93-1.1]	7.67E-01
0.987[0.92-1.06]	7.17E-01
0.983[0.915-1.06]	6.40E-01
1.02[0.915-1.14]	7.23E-01
0.919[0.844-1]	5.33E-02
1.03[0.955-1.1]	4.76E-01
0.953[0.889-1.02]	1.83E-01
0.97[0.878-1.07]	5.56E-01
0.938[0.772-1.14]	5.23E-01
1.06[0.976-1.15]	1.67E-01
1.06[0.97-1.17]	1.87E-01
0.99[0.89-1.1]	8.53E-01

Table S5.Distinct Complement Protein Profiles in AMD PRS and Smoking Status Groupings

Complement Proteins	AMD	Control	OR(AMD vs Control)
C1QBP	0.16	0.12	1.05
C1RL	0.00	-0.01	1.09
C1R	0.02	0.01	1.00
SERPING1	0.03	0.00	1.43
CFHR2	-0.03	-0.10	1.09
CFI	0.02	0.00	0.72
CFHR5	0.04	-0.02	1.28
CFB	0.01	-0.01	0.81
CFHR4	-0.01	-0.10	1.14
C9	0.03	-0.05	1.10
C2	0.02	-0.02	1.30
CD55	0.09	0.02	1.32
CD46	0.10	0.02	1.24
CFD	0.05	0.01	0.92
C5	0.01	0.00	1.04
C3	0.05	0.03	0.91
C1S	0.01	0.00	0.98
C7	0.04	-0.01	0.87
CFH	0.03	-0.01	1.06
FCN2	-0.03	-0.05	1.17
FCN1	0.03	-0.01	0.99
MBL2	-0.11	-0.07	1.00
CR2	-0.02	-0.01	1.10
CLU	0.04	0.03	1.05
CR1	0.03	0.01	0.90
MASP1	0.01	-0.01	1.03
C4BPB	0.10	0.01	1.17
CFP	-0.01	0.00	0.83

5%CI(AMD vs Control)	95%CI(AMD vs Control)	P(AMD vs Control)	Never smoked
0.94	1.16	3.86E-01	0.11
0.73	1.63	6.75E-01	-0.01
0.64	1.50	9.86E-01	0.01
0.94	2.18	9.41E-02	-0.02
0.95	1.26	2.13E-01	-0.10
0.48	1.06	9.69E-02	-0.01
1.06	1.56	1.26E-02	-0.05
0.63	1.03	9.00E-02	-0.03
1.02	1.28	1.77E-02	-0.09
0.95	1.28	2.21E-01	-0.04
0.96	1.77	9.47E-02	-0.02
1.03	1.69	2.75E-02	0.02
0.99	1.56	6.14E-02	0.02
0.62	1.33	6.46E-01	0.00
0.61	1.74	8.84E-01	0.00
0.75	1.10	3.42E-01	0.03
0.67	1.44	9.36E-01	-0.01
0.68	1.11	2.63E-01	-0.02
0.74	1.51	7.63E-01	-0.02
0.99	1.39	6.77E-02	-0.06
0.87	1.14	9.17E-01	-0.03
0.93	1.08	9.70E-01	-0.09
0.96	1.27	1.73E-01	0.01
0.80	1.33	6.76E-01	0.04
0.72	1.13	3.72E-01	0.03
0.77	1.38	8.48E-01	-0.01
0.99	1.39	6.24E-02	0.05
0.57	1.19	3.25E-01	0.01

Ever smoked	OR(Ever smoked vs Never smoked)	5%CI(Ever smoked vs Never smoked)
0.13	0.99	0.96
-0.01	1.06	0.95
0.01	0.96	0.85
0.01	1.04	0.92
-0.10	2.16	2.07
0.00	1.02	0.92
0.00	1.47	1.40
0.00	0.68	0.63
-0.11	1.43	1.38
-0.05	0.97	0.93
-0.02	1.35	1.24
0.03	1.00	0.92
0.03	1.08	1.01
0.01	0.97	0.87
0.00	0.94	0.81
0.03	0.91	0.86
0.00	0.99	0.89
-0.01	0.90	0.84
0.00	0.76	0.69
-0.04	1.01	0.96
0.01	0.97	0.94
-0.06	1.00	0.98
-0.02	0.97	0.93
0.02	0.99	0.92
0.00	0.98	0.92
-0.01	1.08	0.99
-0.01	1.07	1.02
-0.01	0.54	0.49

95%CI(Ever smoked vs Never smoked)	*P* (Ever smoked vs Never smoked)	Low PRS	High PRS
1.03	6.42E-01	0.12	0.12
1.19	2.76E-01	-0.01	-0.01
1.08	4.67E-01	0.01	0.01
1.17	5.34E-01	0.00	0.00
2.25	4.54E-267	-0.23	0.02
1.14	7.01E-01	0.00	0.00
1.56	2.89E-43	-0.06	0.01
0.73	1.36E-28	0.01	-0.03
1.47	1.15E-109	-0.20	0.00
1.01	1.45E-01	-0.04	-0.05
1.47	2.43E-12	-0.03	-0.01
1.07	9.01E-01	0.02	0.02
1.15	2.79E-02	0.02	0.03
1.09	6.54E-01	0.01	0.01
1.08	3.72E-01	0.00	0.00
0.96	2.21E-04	0.04	0.02
1.10	8.02E-01	0.00	0.00
0.96	2.68E-03	-0.01	-0.02
0.84	4.45E-08	0.00	-0.01
1.06	6.80E-01	-0.05	-0.04
1.01	1.99E-01	0.00	-0.01
1.02	8.69E-01	-0.07	-0.07
1.01	1.95E-01	-0.01	-0.01
1.06	7.43E-01	0.03	0.03
1.05	5.64E-01	0.01	0.01
1.17	7.27E-02	-0.01	0.00
1.12	5.18E-03	0.01	0.02
0.61	2.40E-29	0.01	-0.02

OR(High PRS vs Low PRS)	5%CI(High PRS vs Low PRS)	95%CI(High PRS vs Low PRS)
1.02	0.99	1.06
1.09	0.97	1.22
1.11	0.99	1.26
1.96	1.74	2.22
1.00	0.96	1.04
1.26	1.13	1.42
1.22	1.16	1.30
1.27	1.19	1.37
1.00	0.97	1.03
1.03	0.99	1.08
0.98	0.90	1.07
0.99	0.92	1.08
1.05	0.98	1.12
1.01	0.90	1.14
1.10	0.94	1.28
0.93	0.88	0.98
1.19	1.07	1.33
1.02	0.95	1.10
1.11	1.00	1.23
1.13	1.08	1.19
1.13	1.08	1.17
1.03	1.01	1.05
0.93	0.89	0.97
0.92	0.86	0.99
0.85	0.79	0.90
0.96	0.88	1.05
0.81	0.77	0.85
0.75	0.68	0.83

P (High PRS vs Low PRS)	Low PRS and never smoked	Low PRS and ever smoked
1.69E-01	0.11	0.13
1.59E-01	-0.01	-0.01
7.97E-02	0.01	0.01
2.51E-27	-0.02	0.01
8.39E-01	-0.22	-0.23
6.22E-05	-0.01	0.01
1.59E-12	-0.09	-0.03
1.76E-11	-0.01	0.02
8.40E-01	-0.20	-0.20
1.38E-01	-0.04	-0.04
6.91E-01	-0.03	-0.03
8.65E-01	0.02	0.03
2.05E-01	0.01	0.02
8.37E-01	0.00	0.02
2.18E-01	0.00	0.00
4.41E-03	0.04	0.04
1.54E-03	-0.01	0.00
6.26E-01	-0.01	0.00
5.39E-02	-0.01	0.01
6.15E-07	-0.07	-0.04
6.91E-09	-0.03	0.01
1.58E-02	-0.10	-0.05
3.42E-04	0.01	-0.02
2.39E-02	0.04	0.03
7.29E-07	0.03	0.00
3.72E-01	-0.01	-0.01
1.18E-16	0.04	-0.01
5.74E-08	0.02	0.01

High PRS and never smoked	High PRS and ever smoked
0.12	0.12
0.00	-0.01
0.01	0.01
-0.01	0.01
0.02	0.02
0.00	0.00
-0.01	0.03
-0.05	-0.03
0.01	-0.01
-0.04	-0.05
-0.01	-0.01
0.01	0.03
0.02	0.03
0.00	0.01
0.00	0.00
0.03	0.02
-0.01	0.00
-0.02	-0.02
-0.02	-0.01
-0.06	-0.03
-0.03	0.00
-0.09	-0.06
0.01	-0.03
0.04	0.02
0.03	0.00
0.00	0.00
0.06	0.00
-0.01	-0.02

OR(Low PRS and ever smoked vs Low PRS and never smoked)
1.05
1.17
1.16
2.11
0.99
1.39
1.29
1.28
1.01
1.03
0.99
0.96
1.03
1.02
1.28
0.97
1.25
1.05
1.18
1.13
1.13
1.04
0.93
0.93
0.85
0.95
0.82
0.75

5%CI (Low PRS and ever smoked vs Low PRS and never smoked)	95%CI(Low PRS and ever smoked vs Low PRS and never smoked)
1.00	1.10
0.99	1.38
0.98	1.38
1.78	2.51
0.94	1.04
1.18	1.63
1.20	1.40
1.16	1.42
0.97	1.06
0.97	1.10
0.88	1.10
0.86	1.07
0.94	1.14
0.86	1.21
1.03	1.59
0.90	1.05
1.07	1.45
0.94	1.16
1.02	1.36
1.05	1.21
1.06	1.20
1.01	1.07
0.88	0.99
0.85	1.03
0.77	0.93
0.84	1.07
0.76	0.88
0.65	0.87

P (Low PRS and ever smoked vs Low PRS and never smoked)	OR(High PRS and never smoked vs Low PRS and never smoked)	5%CI(High PRS and never smoked vs Low PRS and never smoked)
6.44E-02	1.02	0.97
6.44E-02	1.17	0.97
7.76E-02	1.02	0.85
1.43E-17	1.17	0.96
6.73E-01	2.14	2.00
6.96E-05	1.15	0.97
6.46E-11	1.61	1.47
5.58E-07	0.68	0.61
6. 17E-01	1.47	1.40
3.65E-01	0.96	0.89
7.92E-01	1.34	1.18
4.31E-01	0.96	0.85
5.07E-01	1.07	0.95
8.08E-01	1.00	0.83
2.64E-02	1.14	0.91
4.44E-01	0.96	0.89
5. 18E-03	1.06	0.89
3.93E-01	0.94	0.84
2.76E-02	0.81	0.69
5.21E-04	1.01	0.94
4.33E-05	0.97	0.91
1.55E-02	1.01	0.98
2.75E-02	0.99	0.92
1.85E-01	1.01	0.91
6.81E-04	1.00	0.90
3.61E-01	1.06	0.93
4.53E-08	1.09	1.01
9.57E-05	0.57	0.49

95%CI(High PRS and never smoked vs Low PRS and never smoked)	*P* (High PRS and never smoked vs Low PRS and never smoked)	OR(High PRS and ever smoked vs Low PRS and never smoked)	5%CI(High PRS and ever smoked vs Low PRS and never smoked)
1.08	4.26E-01	1.02	0.97
1.40	9.53E-02	1.18	1.00
1.21	8.51E-01	1.07	0.90
1.42	1.11E-01	2.05	1.73
2.29	1.55E-102	2.17	2.04
1.38	1.15E-01	1.32	1.12
1.76	5.99E-25	1.84	1.70
0.76	2.06E-11	0.85	0.77
1.55	1.41E-49	1.39	1.33
1.03	2.36E-01	1.00	0.94
1.54	1.74E-05	1.33	1.18
1.09	5.10E-01	0.99	0.88
1.19	2.55E-01	1.13	1.02
1.20	9.98E-01	0.98	0.83
1.44	2.66E-01	1.05	0.84
1.04	3.25E-01	0.85	0.79
1.25	5.16E-01	1.19	1.02
1.06	3.01E-01	0.92	0.83
0.95	1.08E-02	0.85	0.73
1.09	7.67E-01	1.14	1.07
1.03	3.63E-01	1.09	1.03
1.05	4.21E-01	1.03	0.99
1.05	7.12E-01	0.90	0.85
1.12	8.96E-01	0.90	0.81
1.10	9.52E-01	0.84	0.76
1.21	3.89E-01	1.04	0.92
1.18	3.26E-02	0.87	0.81
0.68	4.56E-11	0.40	0.34

95%CI(High PRS and ever smoked vs Low PRS and never smoked)	*P* (High PRS and ever smoked vs Low PRS and never smoked)
1.08	3.56E-01
1.39	5.11E-02
1.27	4.46E-01
2.44	3.02E-16
2.32	3.36E-130
1.55	9.26E-04
2.00	1.42E-47
0.94	1.70E-03
1.46	1.94E-49
1.06	9.33E-01
1.50	5.52E-06
1.11	8.29E-01
1.25	1.52E-02
1.16	8.12E-01
1.32	6.44E-01
0.91	1.55E-05
1.39	2.65E-02
1.03	1.39E-01
0.98	2.46E-02
1.22	1.06E-04
1.15	2.79E-03
1.06	1.04E-01
0.96	7.64E-04
1.00	4.57E-02
0.92	1.71E-04
1.17	5.40E-01
0.93	8.76E-05
0.47	4.08E-29

TableS6.Two-sample Mendelian randomization results between complement proteins and AMD or Ever

Exposure	Outcome	method	nsnp	BETA	Causal_SE	Causal _*P*
C1R	AMD	Weighted median	28	3.59E-03	2.66E-03	1.77E-01
C1RL	AMD	Inverse variance weigh	57	-3.57E-04	8.80E-04	6.85E-01
C1S	AMD	Weighted median	15	-4.21E-04	2.74E-03	8.78E-01
C4BPB	AMD	MR Egger	24	-2.47E-03	7.05E-03	7.29E-01
C7	AMD	Inverse variance weigh	26	9.46E-06	1.52E-03	9.95E-01
CD46	AMD	Weighted median	6	-4.80E-03	9.89E-03	6.27E-01
CD55	AMD	Weighted median	57	-1.17E-03	1.91E-03	5.39E-01
CFB	AMD	Weighted median	98	2.31E-03	2.15E-03	2.82E-01
CFD	AMD	Weighted median	10	4.98E-02	6.94E-03	7.44E-13
CFH	AMD	Weighted median	29	-4.15E-03	2.98E-03	1.64E-01
CFHR2	AMD	MR Egger	159	1.03E-03	1.64E-03	5.33E-01
CFHR4	AMD	Weighted median	192	4.48E-03	8.33E-04	7.74E-08
CFHR5	AMD	Weighted median	39	3.86E-03	1.37E-03	4.86E-03
CFI	AMD	MR Egger	22	1.20E-03	6.62E-03	8.59E-01
CLU	AMD	Inverse variance weigh	8	9.41E-04	3.05E-03	7.58E-01
CR1	AMD	Inverse variance weigh	16	-1.49E-04	1.90E-03	9.38E-01
CR2	AMD	Weighted median	8	-1.00E-02	6.08E-03	9.85E-02
FCN1	AMD	Inverse variance weigh	65	-2.42E-03	1.59E-03	1.27E-01
FCN2	AMD	Inverse variance weigh	109	2.24E-03	1.24E-03	7.10E-02
MASP1	AMD	MR Egger	30	-6.65E-04	7.21E-03	9.27E-01
MBL2	AMD	MR Egger	210	1.76E-03	7.53E-04	2.06E-02
SERPING	AMD	Weighted median	43	1.92E-03	2.70E-03	4.77E-01
C1R	Ever smok	Inverse variance weigh	42	2.40E-02	1.22E-02	5.00E-02
C1RL	Ever smok	MR Egger	58	3.45E-02	1.09E-02	2.55E-03
C1S	Ever smok	Inverse variance weigh	30	2.08E-03	1.09E-02	8.49E-01
C5	Ever smok	Wald ratio	1	1.44E-02	3.65E-02	6.93E-01
C7	Ever smok	Inverse variance weigh	21	-4.51E-03	1.39E-02	7.45E-01
C9	Ever smok	Inverse variance weigh	3	-1.03E-02	4.74E-02	8.29E-01
CD46	Ever smok	Weighted median	17	-8.67E-04	1.83E-02	9.62E-01
CD55	Ever smok	Inverse variance weigh	50	-3.64E-03	9.08E-03	6.88E-01
CFB	Ever smok	Weighted median	63	-4.59E-02	1.17E-02	9.16E-05
CFD	Ever smok	MR Egger	20	1.11E-01	3.19E-02	2.65E-03
CFH	Ever smok	Inverse variance weigh	31	-2.90E-03	1.15E-02	8.01E-01
CFHR2	Ever smok	Weighted median	173	2.95E-03	5.41E-03	5.86E-01
CFHR4	Ever smok	Weighted median	185	1.02E-02	5.20E-03	5.05E-02
CFHR5	Ever smok	Weighted median	61	-2.48E-03	9.72E-03	7.98E-01
CFI	Ever smok	Inverse variance weigh	17	1.33E-02	2.03E-02	5.13E-01
CR1	Ever smok	Weighted median	25	1.20E-02	1.52E-02	4.31E-01
CR2	Ever smok	Inverse variance weigh	14	-5.50E-04	1.95E-02	9.78E-01
FCN1	Ever smok	Inverse variance weigh	73	-6.42E-03	8.56E-03	4.53E-01
FCN2	Ever smok	Weighted median	173	-5.99E-03	6.38E-03	3.48E-01
MASP1	Ever smok	Inverse variance weigh	3	1.72E-02	4.62E-02	7.09E-01
MBL2	Ever smok	Inverse variance weigh	278	6.75E-03	3.29E-03	4.05E-02
SERPING	Ever smok	Weighted median	85	-1.46E-02	9.41E-03	1.21E-01

smoked

Presso-Outlier-corrected_BETA	Presso-Outlier-corrected_SE	Presso-Outlier-corrected_*P*
4.37E-04	2.17E-03	0.84
1.37E-03	2.23E-03	0.55
8.17E-03	3.56E-03	0.03
5.54E-04	9.20E-03	0.96
-5.19E-04	1.64E-03	0.75
5.63E-03	2.18E-03	0.01
-9.02E-03	3.10E-03	0.01
3.71E-03	8.10E-04	0.00
4.90E-03	7.92E-04	0.00
4.00E-03	1.58E-03	0.02
-1.11E-02	4.09E-03	0.01
-1.13E-02	6.46E-03	0.13
2.14E-02	3.19E-03	0.00
1.09E-03	2.21E-03	0.62

Q	Q_df	Q_pval	Egger_intercept	Pleiotropy_SE	Pleiotropy_*P*	FDR
47.36	27	9.04E-03	-1.06E-03	9.54E-04	0.28	4.32E-01
52.39	56	6.12E-01	-4.34E-04	4.10E-04	0.29	9.81E-01
27.35	14	1.74E-02	-6.97E-04	1.30E-03	0.60	9.82E-01
55.93	23	1.45E-04	2.53E-03	1.06E-03	0.03	9.81E-01
30.23	25	2.16E-01	1.02E-03	1.05E-03	0.34	9.95E-01
23.48	5	2.73E-04	2.82E-04	4.57E-03	0.95	9.81E-01
148.21	56	2.86E-10	-1.65E-04	8.06E-04	0.84	9.12E-01
603.79	97	3.19E-74	-9.70E-05	8.41E-04	0.91	6.21E-01
19.38	9	2.21E-02	-1.43E-03	2.54E-03	0.59	1.64E-11
127.16	28	1.36E-14	-6.60E-04	1.68E-03	0.70	4.32E-01
966.39	158	3.43E-116	2.16E-03	9.47E-04	0.02	9.12E-01
1200.09	191	9.80E-146	-9.49E-04	9.79E-04	0.33	8.51E-07
301.65	38	9.09E-43	1.27E-03	1.42E-03	0.38	3.57E-02
57.90	21	2.64E-05	-3.47E-03	1.28E-03	0.01	9.82E-01
7.54	7	3.75E-01	-8.26E-04	9.99E-04	0.44	9.81E-01
19.75	15	1.82E-01	2.35E-03	1.22E-03	0.07	9.82E-01
32.73	7	2.97E-05	-3.05E-03	2.96E-03	0.34	3.61E-01
60.71	64	5.94E-01	-7.63E-05	5.05E-04	0.88	4.00E-01
109.52	108	4.41E-01	4.23E-04	4.64E-04	0.36	3.12E-01
53.87	29	3.35E-03	3.47E-03	1.16E-03	0.01	9.82E-01
232.54	209	1.26E-01	-8.47E-04	3.03E-04	0.01	1.13E-01
70.48	42	3.85E-03	-3.34E-04	7.69E-04	0.67	9.12E-01
55.46	41	6.53E-02	1.92E-03	2.62E-03	0.47	1.85E-01
66.02	57	1.93E-01	-5.38E-03	1.84E-03	0.01	1.94E-02
29.69	29	4.30E-01	-6.38E-05	2.93E-03	0.98	9.34E-01
						9.34E-01
19.78	20	4.72E-01	-6.55E-03	5.50E-03	0.25	9.34E-01
1.00	2	6.06E-01	-6.21E-03	6.94E-03	0.54	9.34E-01
29.73	16	1.95E-02	-3.38E-03	8.42E-03	0.69	9.78E-01
51.07	49	3.92E-01	8.71E-04	2.64E-03	0.74	9.34E-01
141.54	62	3.71E-08	-5.60E-04	2.82E-03	0.84	2.02E-03
21.09	19	3.32E-01	-8.21E-03	3.81E-03	0.04	1.94E-02
29.59	30	4.87E-01	3.48E-03	3.30E-03	0.30	9.34E-01
227.00	172	3.14E-03	3.18E-04	1.98E-03	0.87	9.34E-01
248.70	184	1.05E-03	7.40E-04	2.03E-03	0.72	1.85E-01
90.49	60	6.67E-03	6.74E-04	2.23E-03	0.76	9.34E-01
15.91	16	4.59E-01	4.32E-03	5.66E-03	0.46	9.34E-01
50.62	24	1.18E-03	-5.74E-03	5.83E-03	0.33	9.34E-01
13.03	13	4.46E-01	-3.82E-03	3.71E-03	0.32	9.78E-01
88.82	72	8.70E-02	1.42E-04	2.03E-03	0.94	9.34E-01
203.70	172	4.95E-02	1.64E-03	1.21E-03	0.18	9.34E-01
0.34	2	8.44E-01	6.79E-03	1.68E-02	0.76	9.34E-01
293.49	277	2.37E-01	5.21E-04	1.26E-03	0.68	1.85E-01
136.22	84	2.74E-04	1.50E-03	2.77E-03	0.59	3.80E-01

TableS7.Colocalization results between complement proteins and AMD or Ever smoked

Exposure	Outcome	nsnps	PP.H0.abf	PP.H1.abf	PP.H2.abf	PP.H3.abf
CFD	AMD	24	9.11E-88	1.95E-87	7.17E-04	5.35E-04
CLU	AMD	9	1.98E-29	2.24E-33	9.93E-01	1.06E-04
C1R	AMD	3	1.07E-07	6.44E-12	9.96E-01	5.60E-05
CFI	AMD	29	2.35E-191	2.92E-184	8.04E-11	2.07E-10
C5	Ever smok	8	5.05E-22	2.66E-26	9.98E-01	5.03E-05
CR1	Ever smok	66	6.58E-45	2.56E-47	9.90E-01	3.84E-03
CFB	Ever smok	177	3.44E-46	6.76E-46	2.36E-01	4.64E-01
C9	Ever smok	8	3.76E-21	1.88E-25	9.94E-01	4.34E-05
C3	AMD	2	1.10E-43	1.18E-35	9.34E-12	5.12E-16
C1RL	AMD	5	3.97E-25	1.85E-29	9.96E-01	4.22E-05
C4BPB	AMD	28	2.52E-97	5.83E-98	8.07E-01	1.87E-01
FCN2	Ever smok	191	2.10E-45	3.09E-47	9.80E-01	1.44E-02
C9	AMD	7	7.27E-25	3.11E-27	9.89E-01	4.22E-03
CD46	Ever smok	30	6.88E-182	5.33E-185	9.97E-01	7.71E-04
CR2	AMD	28	2.91E-127	4.65E-127	3.83E-01	6.12E-01
CFH	Ever smok	33	2.48E-149	1.14E-152	9.96E-01	4.53E-04
CD55	Ever smok	62	5.61E-45	3.74E-48	9.90E-01	6.52E-04
MASP1	Ever smok	6	5.98E-17	1.30E-21	9.98E-01	1.93E-05
CFHR2	Ever smok	158	1.08E-45	7.21E-48	9.77E-01	6.49E-03
C1S	AMD	2	5.79E-06	1.65E-10	9.87E-01	1.50E-05
C2	AMD	62	9.89E-190	2.50E-164	3.95E-26	1.00E+00
C3	Ever smok	1	1.05E-08	4.45E-14	9.96E-01	0.00E+00
MBL2	Ever smok	283	1.05E-45	4.13E-48	9.85E-01	3.85E-03
C1RL	Ever smok	13	2.42E-26	5.90E-30	9.82E-01	2.22E-04
CFP	Ever smok	72	2.13E-19	1.12E-22	9.96E-01	5.21E-04
CFD	Ever smok	38	7.04E-46	3.23E-49	9.66E-01	4.09E-04
CFB	AMD	249	0.00E+00	1.4612157000	9.37E-54	1.00E+00
CFHR5	AMD	93	0.00E+00	1.1125369293	1.93E-259	9.90E-04
SERPING	Ever smok	120	2.16E-45	9.36E-48	9.78E-01	4.22E-03
C1S	Ever smok	10	7.82E-13	8.36E-17	9.95E-01	1.02E-04
FCN1	Ever smok	79	7.13E-45	5.64E-47	9.87E-01	7.80E-03
CR2	Ever smok	34	6.88E-87	3.59E-90	9.93E-01	5.13E-04
C2	Ever smok	61	8.26E-46	8.43E-46	2.67E-01	2.72E-01
CD46	AMD	11	1.27E-25	1.14E-27	1.08E-01	7.70E-05
C7	AMD	78	1.53E-298	1.54E-295	9.92E-04	9.99E-01
C7	Ever smok	67	3.92E-45	2.06E-48	9.95E-01	5.18E-04
CFI	Ever smok	23	4.90E-44	1.27E-47	9.93E-01	2.50E-04
CFHR4	AMD	162	0.00E+00	1.1126998102	9.33E-158	1.00E+00
CD55	AMD	64	2.66E-273	4.90E-273	3.51E-01	6.47E-01
CFH	AMD	62	0.00E+00	6.12E-273	3.18E-270	1.00E+00
CFHR2	AMD	161				
C1R	Ever smok	5	3.05E-12	4.62E-16	9.82E-01	1.31E-04
CFHR4	Ever smok	159	9.31E-46	1.44E-47	9.20E-01	1.41E-02
MBL2	AMD	166				
SERPING	AMD	59	3.36E-132	4.99E-135	9.88E-01	1.46E-03
CR1	AMD	44	1.10E-213	1.48E-212	6.91E-02	9.31E-01
FCN1	AMD	67	9.81E-308	1.1366790401	9.78E-01	4.80E-03
MASP1	AMD	35	7.81E-165	2.49E-166	4.41E-02	4.49E-04

PP.H4.abf
9.99E-01
6.93E-03
4.11E-03
1.00E+00
2.23E-03
5.87E-03
3.00E-01
6.24E-03
1.00E+00
4.20E-03
6.57E-03
5.67E-03
6.84E-03
2.27E-03
5.59E-03
3.50E-03
8.97E-03
2.32E-03
1.61E-02
1.31E-02
6.79E-15
4.21E-03
1.09E-02
1.78E-02
3.24E-03
3.35E-02
8.54E-30
9.99E-01
1.79E-02
4.57E-03
4.76E-03
6.19E-03
4.61E-01
8.92E-01
4.53E-06
4.23E-03
6.27E-03
9.90E-94
2.51E-03
1.04E-221
1.78E-02
6.61E-02
1.01E-02
3.81E-04
1.71E-02
9.55E-01

Supplementary Figure 1.Manhattan plot of the meta-analysis for AMD GWAS

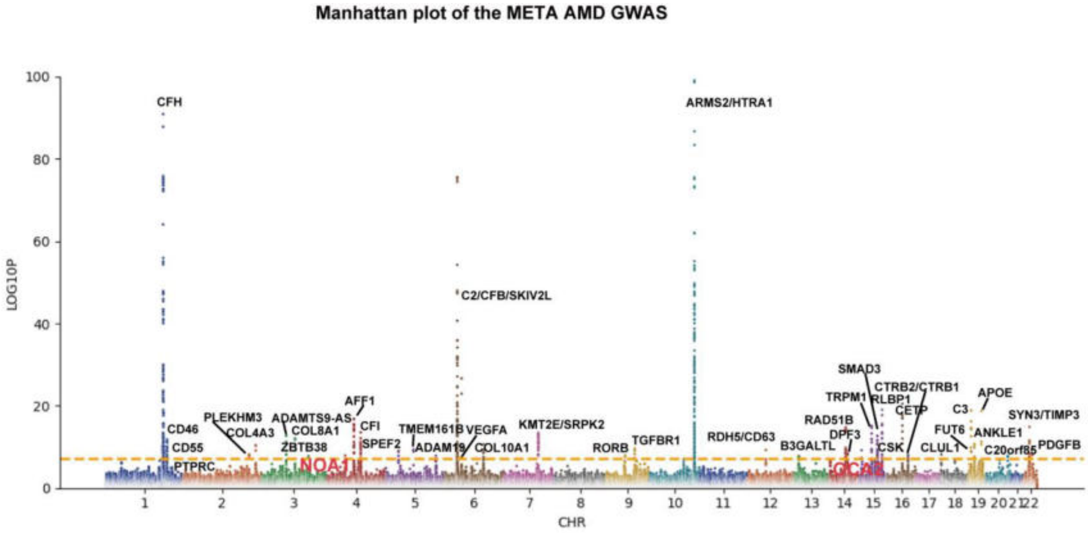

Manhattan plot of the meta-analysis for AMD GWAS. Newly identified loci are highlighted in red.

Supplementary Figure 2. Correlation between UKB AMD BETA and META AMD BETA

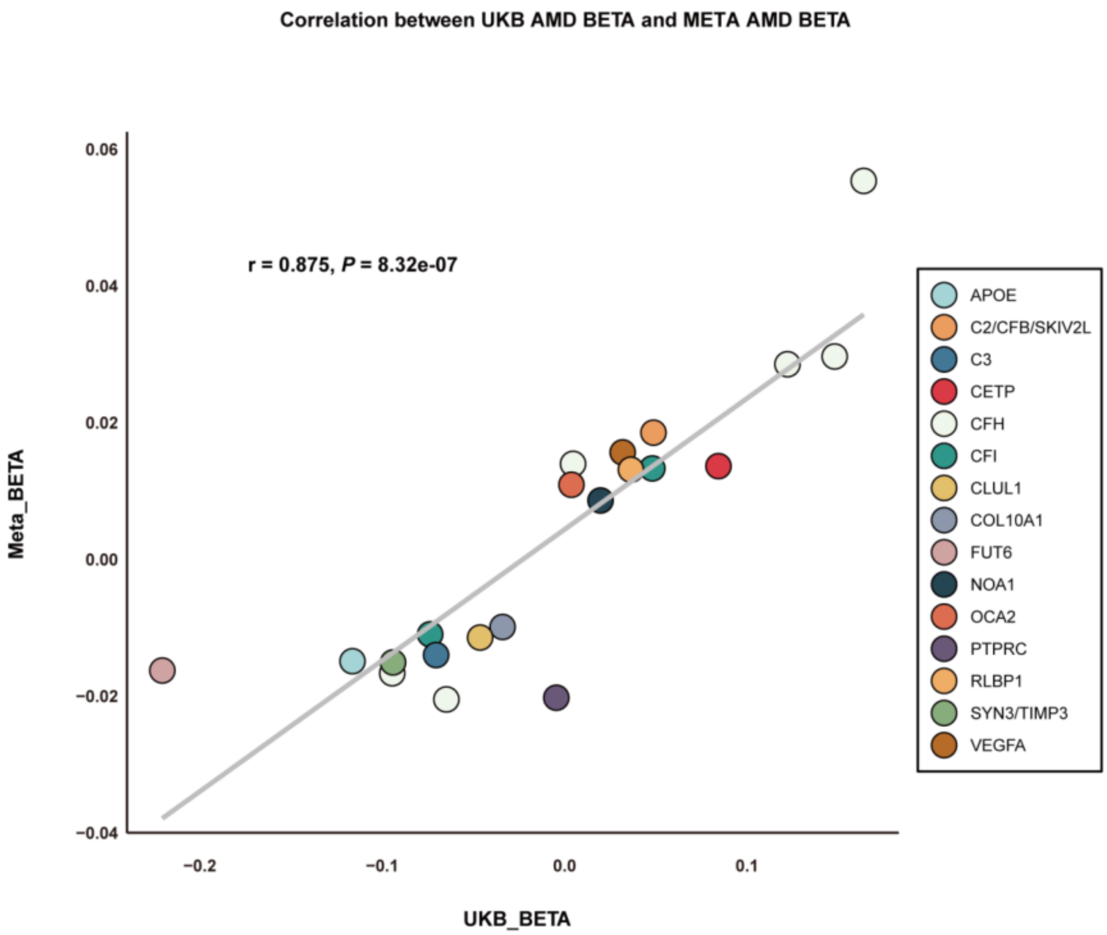

Correlation scatter plot showing a strong positive correlation (r = 0.88, P = 3.82×10^{-7}) between the effect sizes of 21 causal variants in the meta AMD GWAS and the UK Biobank AMD GWAS.

Supplementary Figure 3. ROC Curve of Null Model and Polygenic Risk Score Model

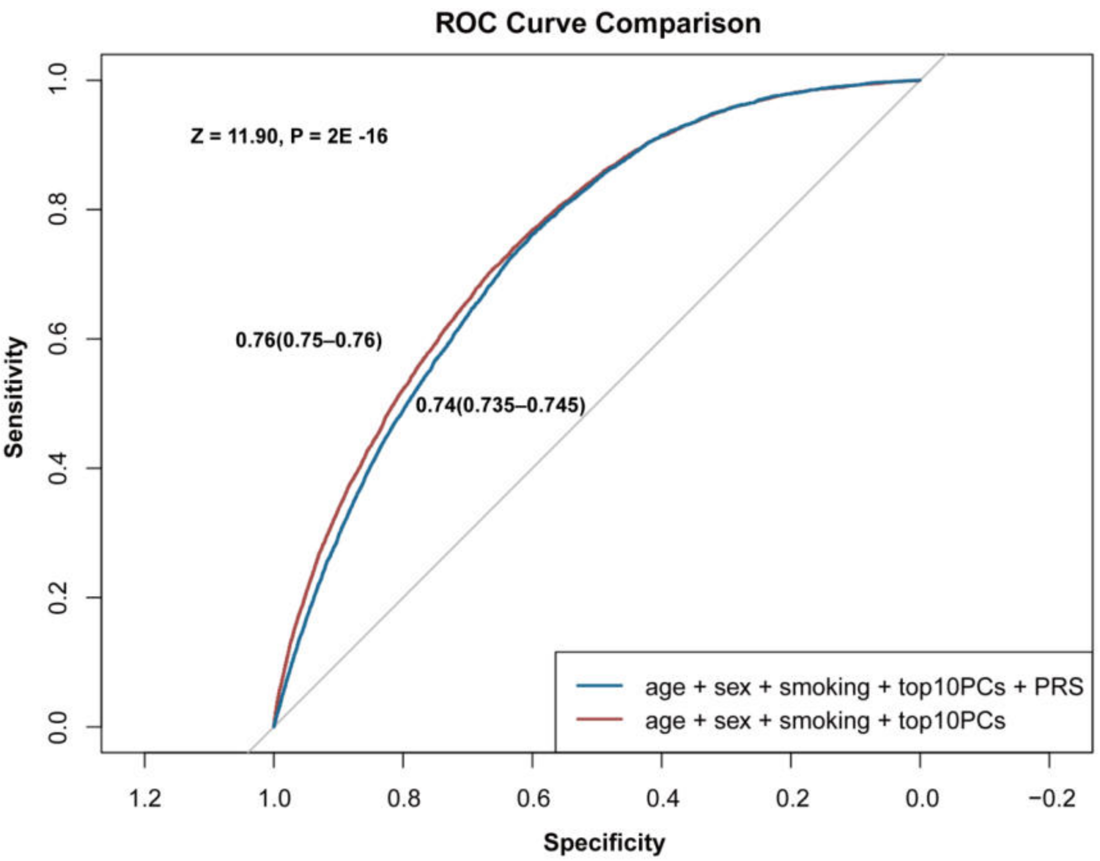

ROC curves comparing model performance. The null model (blue), incorporating age, sex, ever smoked, and the top 10 principal components (PCs), achieved an AUC of 0.74 (95% CI, 0.74–0.75). The addition of the AMD PRS to the traditional risk factors significantly improved the AUC of PRS model (red) to 0.76 (95% CI, 0.75–0.76; Z = 11.90, P = 2×10^{-16}).

Supplementary Figure 4. Bootstrap Distribution of Δ AUC

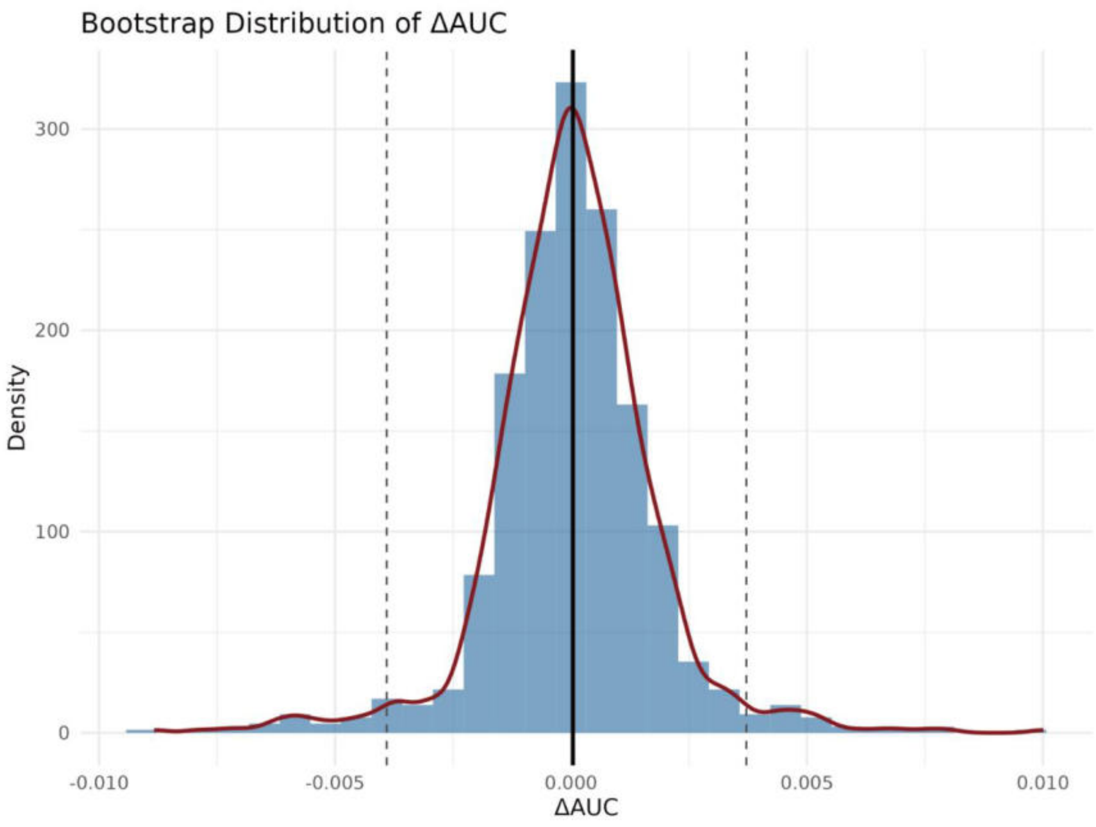

Supplementary Figure 5. Cumulative Incidence of AMD in the UKBB Cohort Under Different Polygenic Risk Score Threshold

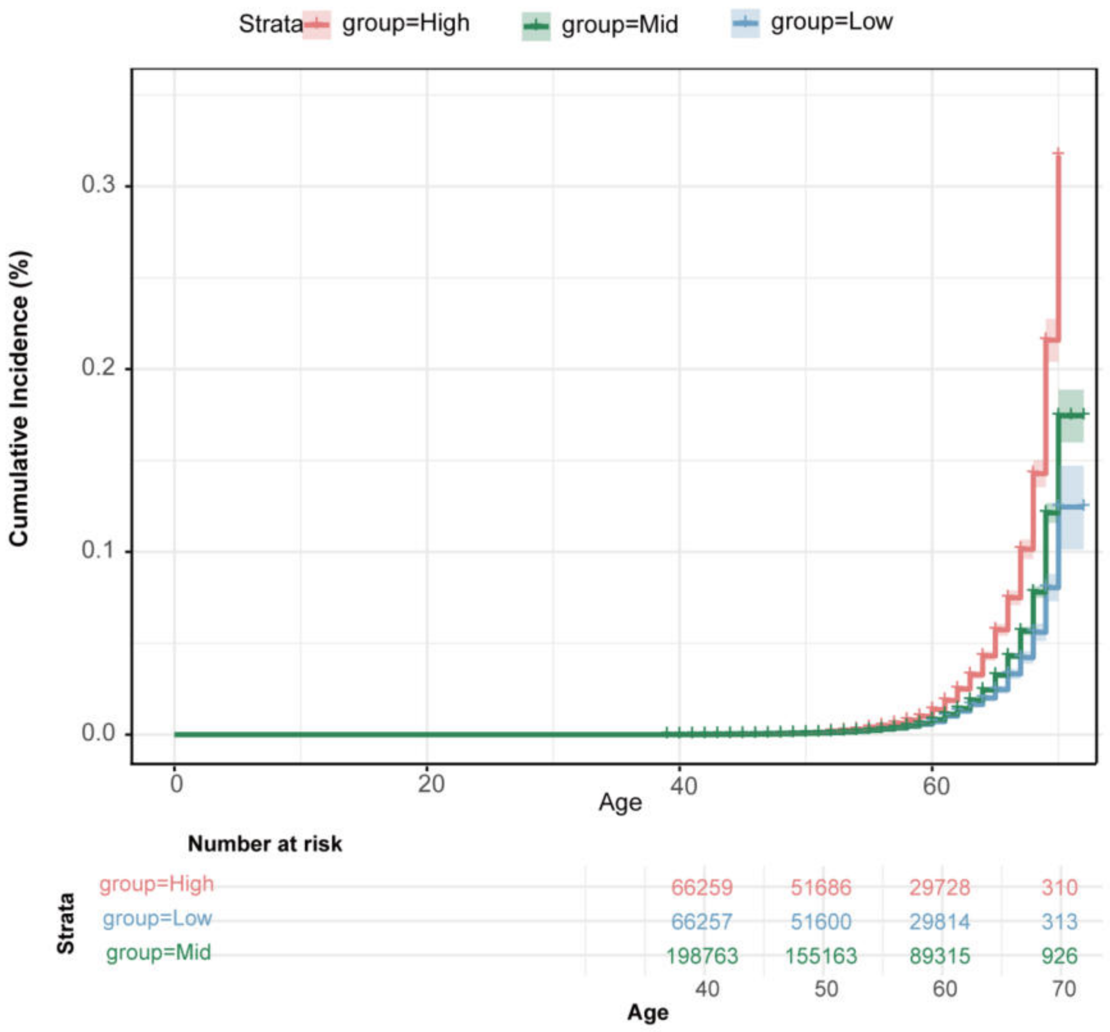

Cumulative incidence of AMD in the UKBB cohort for individuals over 70 years old. The incidence among high-PRS individuals (red) increased significantly to 31.7% (±1.6%), compared to 12.5% (±1.1%) in low-PRS individuals (blue) and 17.4% (±0.7%) in mid-PRS individuals (green).

Supplementary Figure 6. Variant-smoking Models

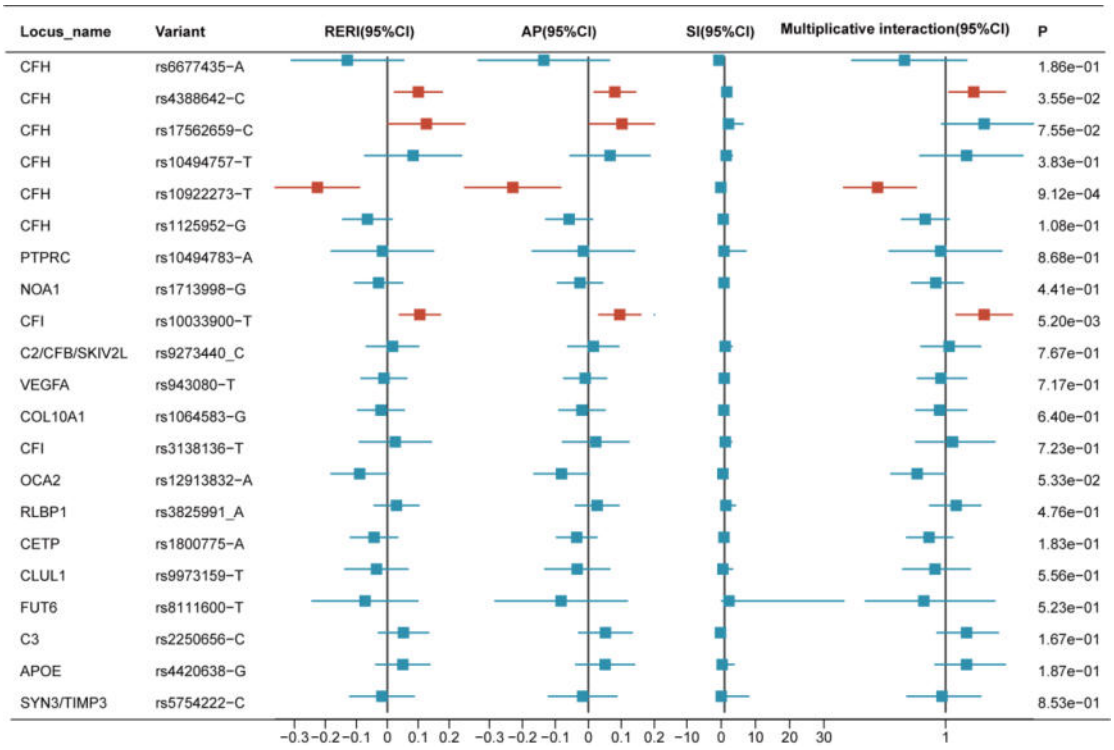

Forest plot illustrates the additive and multiplicative interactions between genetic variants (rs4388642-C, rs10922273-T, and rs17562659-C in CFH locus, as well as rs10033900-T in CFI locus) and ever smoked on AMD risk. Red lines indicate the presence of significant interactions.

Supplementary Figure 7. Summary of Differential Expression Analysis

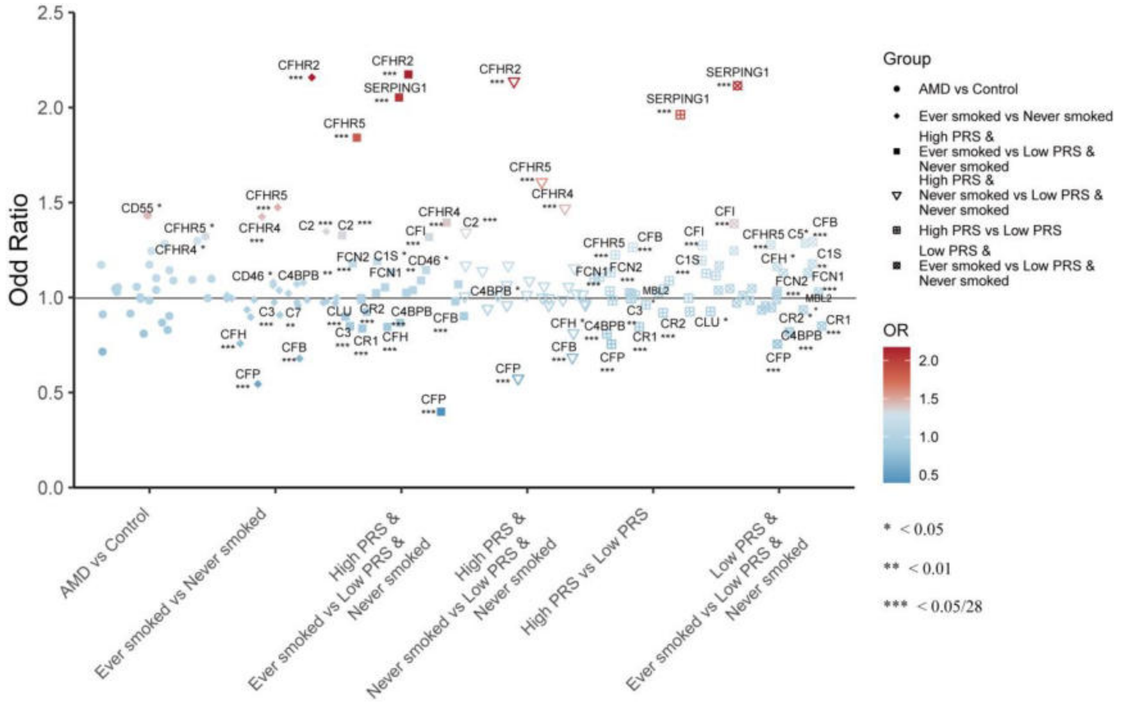

The manhattan plot represents the differential expression (DE) results for six key group comparisons: AMD versus control, ever smoked versus never smoked, high PRS versus low PRS, individuals who have low PRS and ever smoked versus individuals who have low PRS and never smoked, individuals who have high PRS and never smoked vs. individuals who have low PRS and never smoked, and individuals who have high PRS and ever smoked versus individuals who have low PRS and never smoked.

Supplementary Figure 8. Mendelian Randomization and Colocalization Analyses Identify Complement Proteins Associated with AMD

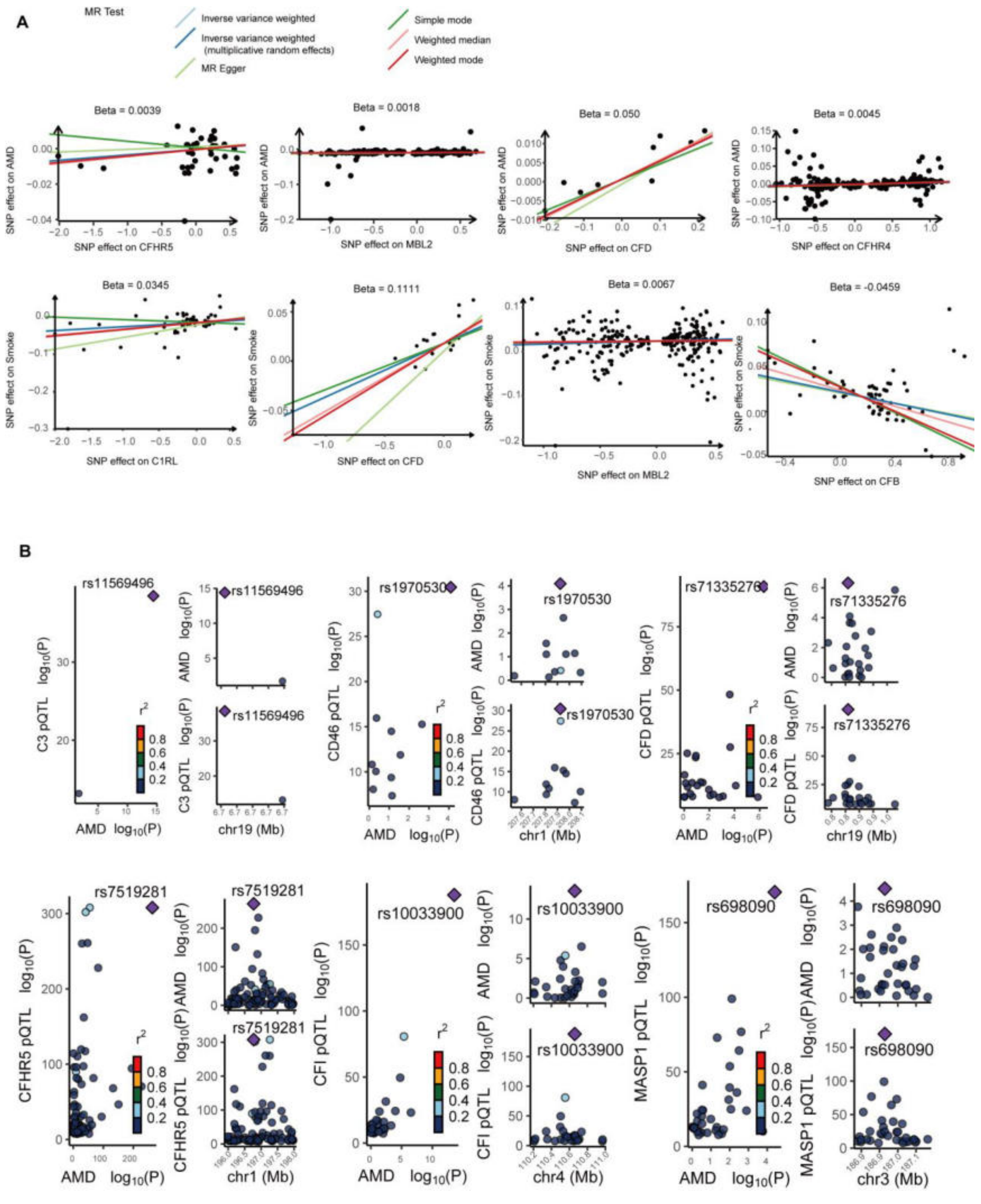

© 2025 Guo J. et al.
